# Discovery of Novel Viruses Associated With the Invasive Cane Toad (*Rhinella marina*) in Its Native and Introduced Ranges

**DOI:** 10.3389/fmicb.2021.733631

**Published:** 2021-09-06

**Authors:** Alice G. Russo, Emma F. Harding, Grace J. H. Yan, Daniel Selechnik, Simon Ducatez, Jayna L. DeVore, Jia Zhou, Roshmi R. Sarma, Yin Peng Lee, Mark F. Richardson, Richard Shine, Lee A. Rollins, Peter A. White

**Affiliations:** ^1^School of Biotechnology and Biomolecular Sciences, University of New South Wales, Sydney, NSW, Australia; ^2^School of Life and Environmental Sciences (SOLES), University of Sydney, Sydney, NSW, Australia; ^3^School of Biological, Earth and Environmental Sciences, University of New South Wales, Sydney, NSW, Australia; ^4^School of Agriculture, Food and Wine, University of Adelaide, Adelaide, SA, Australia; ^5^School of Life and Environmental Sciences, Deakin University, Geelong, VIC, Australia; ^6^Department of Biological Sciences, Macquarie University, Sydney, NSW, Australia

**Keywords:** cane toad, *Rhinella marina*, viral discovery, invasive species, virome

## Abstract

Cane toads (*Rhinella marina*) are notoriously successful invaders: from 101 individuals brought to Australia in 1935, poisonous toads now cover an area >1.2 million km^2^ with adverse effects on native fauna. Despite extensive research on the role of macroparasites in cane toad invasion, viral research is lagging. We compared viral prevalence and diversity between toads in their native range (French Guiana, *n*=25) and two introduced ranges: Australia (*n*=151) and Hawai’i (*n*=10) with a metatranscriptomic and metagenomic approach combined with PCR screening. Australian toads almost exclusively harbor one of seven viruses detected globally. Rhimavirus-A (*Picornaviridae*) exhibited low genetic diversity and likely actively infected 9% of sampled Australian toads extending across ~2,000km of Northern Australia and up to the current invasion front. In native range cane toads, we identified multiple phylogenetically distinct viruses (*Iridoviridae*, *Picornaviridae*, *Papillomaviridae*, and Nackedna-like virus). None of the same viruses was detected in both ranges, suggesting that Australian cane toads have largely escaped the viral infection experienced by their native range counterparts. The novel native range viruses described here are potential biocontrol agents, as Australian toads likely lack prior immunological exposure to these viruses. Overall, our evidence suggests that there may be differences between viruses infecting cane toads in their native vs. introduced ranges, which lays the groundwork for further studies on how these viruses have influenced the toads’ invasion history.

## Introduction

Invasive species – those which are introduced, proliferate, and spread outside their native geographical range – pose an immense threat to global biodiversity (Szabo et al., 2012; Bellard et al., 2016; Doherty et al., 2016). To reduce the environmental impact of invaders, it is essential to understand why some species invade new places more readily than others (Mack et al., 2000; Kolar and Lodge, 2001). One potentially crucial facet of the invasion process is viral infection, which can impede an introduced species’ survival, exacerbate its effects on native ecosystems, or in rare cases facilitate its proliferation (Rua et al., 2011). This means that the characterization of viruses infecting invaders is indispensable to both the understanding and mitigation of their impact.

One of the most successful invasive vertebrates is the cane toad (*Rhinella marina*). Cane toads have been deliberately introduced from their native range in South America at least 40 times to over 20 separate countries since the mid-1800s, mainly by the sugarcane industry for pest control (Zug and Zug, 1979; Easteal, 1981; Acevedo et al., 2016). From 1842 to 1932, cane toads were successively translocated through Martinique, Barbados, Puerto Rico, and Hawai’i. In 1935, 101 Hawai’ian toads were brought to Queensland (QLD), Australia (AU) to control the cane beetle (Easteal, 1981; Turvey, 2013). Despite the low genetic diversity resulting from four sequential population bottlenecks, the founders’ progeny spread rapidly and today occupy over 1.2 million km^2^ of mainland Australia (Urban et al., 2008; Rollins et al., 2015). Cane toad bufotoxin, secreted by the toad during all its life-stages, is fatal to many Australian predators, and population declines in toad-eating animals have occurred since the toads’ introduction (Letnic et al., 2008; Shine, 2010; Brown et al., 2011; Jolly et al., 2015, 2016). Current control strategies have been unable to eradicate the toads or curtail their spread (Tingley et al., 2017). Hence, the complex array of factors underpinning the cane toads’ success must be investigated to both understand and mitigate the toads’ impact.

In an introduced range, viral infection may influence the success of invasive species such as the cane toad. In contrast to the native range, where organisms harbor a wide diversity of viruses with which they have co-evolved for millennia, the introduced range contains only a subset of these viruses due to the inevitable viral and host population bottleneck that comes with introduction. Alternatively, infectious agents may be lost soon after arrival due to low infection rates or absence of secondary hosts. This reduction in viral diversity – “enemy release” – may enhance the proliferation, and hence invasion potential, of an introduced species that now faces fewer population restraints (i.e., regulation of the population by pathogens; Keane and Crawley, 2002; Mitchell and Power, 2003). Enemy release may also permit the invader to downregulate metabolically costly immune responses in favor of dispersal-enhancing traits, conferring a competitive advantage over native species (Brown et al., 2015). In another scenario, viral transmission from invaders to immunologically naïve native hosts can cause disease outbreaks in local wildlife, known as “spill-over.” For example, spill-over from an invasive species occurred when domestic dogs (*Canis lupus familiaris*) transmitted canine distemper virus (*Paramyxoviridae*) to African lions (*Panthera leo*) on a Serengeti reserve, causing the death of one-third of the lion population (Weckworth et al., 2020). The host-switching that accompanies spill-over can also increase disease severity in the new host – often a vulnerable native animal – due to the virus’s lack of adaptation to that new host.

Despite the potentially dramatic consequences, little research exists comparing the viromes of natural and invasive populations of invasive vertebrates (Medd et al., 2018). High throughput metatranscriptomics is an effective method to map an animal’s natural virome (Shi et al., 2018; Wille et al., 2018) and can be extended easily to multiple ranges. Therefore, documenting the virome of the cane toad across many countries with a metatranscriptomic approach can address whether enemy release, from a viral point of view, has accompanied the invasion process. Viral biological control is also a viable option for controlling invasive populations but requires finding suitable viral candidates, for example, European rabbits (*Oryctolagus cuniculus*) have been successfully controlled with myxoma virus (*Poxviridae*) and rabbit hemorrhagic disease virus (*Caliciviridae*) in Australia (Strive and Cox, 2019), while yet-to-be-released cyprinid herpesvirus-3 (*Alloherpesviridae*) remains a promising solution to the carp (*Cyprinus carpio*) invasion of Australian waterways (McColl et al., 2014). Although viral biological control offers a potential way to control cane toads, past studies have failed to find a suitable virus, and the studies lack sequencing data, precluding accurate viral classification (Speare, 1990). Therefore, sequencing-based studies of cane toad viruses are needed.

Lastly, viral discovery in toads will assist future studies, which examine the risk posed to amphibians by viral disease. Amphibians are the most threatened class of vertebrates with 13% of assessed species at risk of extinction (IUCN, 2021), and yet few viruses are known to infect amphibians when compared to mammals and birds. Although pathogens are significant threats to many amphibian species (Hof et al., 2011), the scarcity of viruses known to infect these animals makes it difficult to track viral transmission among declining populations. Crucially, since viruses brought by invasive species such as the cane toad may be transmitted to native species, cane toad viral discovery can assist surveillance of any viral pathogens transmitted to native Australian amphibians, many of which are endangered.

These knowledge gaps warrant further virome studies in both the toads’ introduced and native ranges. In a previous metatranscriptomic study of 16 Australian cane toads; we identified three viral families capable of infecting cane toads (*Picornaviridae*, *Circoviridae*, and *Retroviridae*; Russo et al., 2018). However, the full spectrum of cane toad viruses is likely to extend beyond these three families, especially since previous studies bulk sampling amphibians can identify dozens of new viral species at once (Shi et al., 2018). To address this, in this study, we expanded the sample size and geographical range, and viral detection methods employed in our previous study. We acquired numerous cane toad samples collected for other studies and repurposed these to perform a multi-continental, deep-sequencing survey of the cane toad metavirome (viruses infecting the cane toad, and its associated symbionts). We first aimed to further map the cane toad virome in its introduced range by assessing viral epidemiology and diversity across Australia, and another introduced range (Hawai’i). We also studied the toad’s native range – South America – where we investigated the toad’s natural (“pre-invasive”) virome structure to see if and how it differs from Australia.

## Materials and Methods

### Cane Toad Sample Collection

In order to maximize the likelihood of finding viral sequences, this study used cane toad samples and datasets collected for other studies and repurposed them for viral discovery. To find viruses among different cane toad populations, we sourced cane toad samples previously collected from both their native range [French Guiana (FG), *n*=25], and two countries within their introduced range: AU, *n*=107 and Unites States (Hawai’i, HI) *n*=10. Collection of Australian cane toads took place between May 2014 and December 2018 in the Northern Territory (NT; *n*=7 toads), Queensland (QLD; *n*=51), and Western Australia (WA; *n*=49; Supplementary Table S1). Between August and November 2017, French Guianese toads were collected near Regina and St. George (Supplementary Table S1; DeVore et al., 2020). Hawai’ian cane toads (*n*=10) were collected from two regions on the island of O’ahu in June 2015 (Supplementary Table S1). Toads were collected at night and euthanized by lethal injection. Spleen and/or liver were excised, stored in RNA*later* (Thermo Fisher), and refrigerated until further use. All procedures involving live animals were approved by the University of Sydney Animal Care and Ethics Committee (2014/562), the Deakin University Animal Ethics Committee (AEX04-2014), and the University of Adelaide Animal Ethics Committee (S-2018-056). The collection, transportation and access to genetic resources from French Guiana toads was made under the French Ministère de la Transition Ecologique et Solidaire permit TREL1734890A/1 (19 December 2017) and the arrêté from the Préfet de la Région Guyane APmodif-R03-2017-07-18-006.

### RNA Extraction, Preparation, and Sequencing

Prior to using bulk RNA-Seq to identify viral transcripts, RNA was extracted from either cane toad spleen or liver, which typically exhibit a high viral load in vertebrates. In total, RNA was extracted from 150 tissue samples from 142 individuals. Spleens were flash-frozen in liquid nitrogen before being pummeled with a sterile mortar and pestle. Either spleen pieces or liver samples were then homogenized in TRIzol™ (Thermo Fisher) using a FastPrep-24™ (MP Biosciences) for three rounds of 30s at 6m/s. RNA was then extracted using the RNeasy Kit (Qiagen) with a DNase treatment step. RNA integrity was assessed by either RNA 6000 Nano Kit (Agilent) or Qubit™ RNA XR Assay Kit (Thermo Fisher) prior to RNA-Seq.

RNA-Seq was performed specifically for viral discovery as previously described (Russo et al., 2018). Specifically, RNA extracts from the spleen or liver of 78 cane toads (AU origin, *n*=43, FG origin *n*=25, and HI origin *n*=10) were diluted to equimolar concentrations, and then pooled into eight separate libraries (RM_1–RM_8) based on collection location with each library representing between seven and 12 individual toads (Supplementary Table S1). Prior to sequencing, pooled RNA underwent ribosomal RNA (rRNA) depletion with the Ribo-Zero Gold Kit (Illumina) to remove host rRNA, while simultaneously retaining viral sequences which may not possess a poly(A) tail. Following rRNA removal, cDNA synthesis was performed, and libraries were sequenced on the Illumina HiSeq 2500 platform [2×150bp paired end (PE) reads] to a depth of approximately 50,000,000 reads per library. Sequencing was performed at the Australian Genome Research Facility in Melbourne, AU.

### Preparation and NGS of DNA Samples

We also repurposed DNA-Seq data to look for DNA virus genomes. This DNA, originally extracted for cane toad genome resequencing, was obtained from four French Guianese toad livers (RMF031, RMF042, RMF044, and RMF048; Supplementary Table S1). DNA was extracted using a Gentra PureGene Kit (Qiagen). Genomic DNA was quantified with both the Qubit™ dsDNA BR and HS Assay Kits (Thermo Fisher) using a Qubit® 3.0 Fluorometer (Thermo Fisher). For each sample, 70–100ng of genomic DNA was sonicated to a target size of 350bp using a Q800R sonicator (Qsonica, CT, Unites States). The sonicated DNA was purified with AMPure XP magnetic beads (Beckman Coulter).

For the genomic DNA (*n*=4 samples), per-sample sequencing libraries were prepped using the NEBNext Ultra DNA Library Prep Kit for Illumina (NEB) according to the manufacturer’s protocol. Adaptors were ligated with the NEBNext® Multiple Oligos for Illumina (NEB). The PCR enrichment of adaptor-ligated DNA cycling conditions, denaturation, and annealing/extension cycle steps were repeated for a total of eight cycles. Quantification and size estimation of the libraries were performed on both the Qubit 3.0 Fluorometer and 4200 TapeStation System (Agilent). DNA libraries were treated with Illumina Free Adapter Blocking Reagent (Illumina). The pooled library was pre-sequenced on the MiniSeq Sequencer (2×150bp PE reads; Illumina) to obtain the read distribution of each sample. Each library was then re-pooled to equimolar concentrations, enzymatically treated, denatured, and normalized to 2nM. Finally, the library was sequenced on the NovaSeq 6000 Sequencer (2×150bp PE reads; Illumina) at the Deakin University Genomics Centre, Geelong, AU.

### Collation of Additional Publicly Available RNA-Seq Data to Identify Additional Viral Transcripts

In this study, we also looked for viral sequences in splenic RNA-Seq data generated for immunological studies (Selechnik et al., 2019). This comprised 18 RNA-Seq datasets of HI (*n*=10) and FG origin (*n*=8; BioProject accession PRJNA510261). A cane toad liver transcriptome from a commercial supplier in Denmark was also downloaded, assembled, and annotated for viral sequences (ERR2198610; Chana-Muñoz et al., 2019).

### Raw Data Assembly and Annotation for Virus-Like Sequences

Raw sequencing data were quality examined with FastQC (v0.11.6) prior to processing (Andrews, 2010). Reads from RNA-Seq runs were assembled *de novo* with Trinity (v2.5.1; Haas et al., 2013), which included read adapter and quality trimming with Trimmomatic (Bolger et al., 2014).

DNA sequencing reads were also quality-trimmed with Trimommatic (v0.38) and aligned to the cane toad genome (BioProject accession PRJEB24695) with HISAT2 (v2.1.0; Kim et al., 2015; Edwards et al., 2018). Trimmed DNA reads, which did not map to the host genome, and therefore which might be viral in origin, were extracted with SAMtools (v1.9; Li et al., 2009) and assembled with Megahit (v1.2.2b; Li et al., 2015b).

Annotation of assembled RNA or DNA contigs was performed with DIAMOND (v0.9.24.125) using a BLASTx search against the NCBI *nr* protein database (Buchfink et al., 2015). Sequences annotated as “viral,” but which were probably not viral in origin (i.e., host transcripts, which resembled eukaryotic homologs from large DNA viruses) were manually discerned based on their presence in multiple transcriptomes and removed from further analysis. Remaining viral contigs were classified as derived from putative host-infecting viruses if their BLAST hits were derived from viral families, which usually infect vertebrates. Conversely, viral contigs with BLAST hits from families, which usually infect non-vertebrates were classified as putatively host-associated but not host-infecting (e.g., associated with a toad parasite).

### Annotation of a Novel Iridovirus Transcriptome From the Liver of French Guianese Cane Toads

Transcripts with significant homology (*E*-value<1e^−3^) to iridovirus protein sequences based on BLAST search were extracted from the RM_6 transcriptome (Supplementary Table S4). To identify additional iridovirus transcripts not detected with DIAMOND, all predicted protein sequences from *Cherax quadricarinatus* iridovirus (NCBI:txid2035708; *n*=151) and erythrocytic necrosis virus (ENV; NCBI:txid1543320; *n*=95) were used as queries in a tBLASTn search against the RM_6 transcriptome in Geneious (v10.2; Kearse et al., 2012) and resulting contigs underwent a reverse tBLASTx search against the NCBI *nr* database (October 2019, containing all annotated proteins deposited in GenBank) to eliminate non-specific hits. All putative protein coding open reading frames (ORFs) on remaining contigs were translated in Geneious (v10.2) and subjected to a reverse BLASTp search against the *nr* database to identify which ORFs corresponded to which iridovirus protein, this time with a higher *E*-value cut-off (*E*-value<10) to catch more distantly related viral sequences. ORFs with no match in the *nr* database, but which were encoded on the same transcript as an iridovirus ORF (indicating that they were also viral in origin) were annotated as “hypothetical proteins.”

### PCR/RT-PCR Amplification of Partial Viral Transcripts From Cane Toad Liver or Spleen

When putative vertebrate-specific viral transcripts were detected in an RNA-Seq library containing multiple toads, all livers or spleens from individual cane toads comprising that library were first screened for viral genomes *via* RT-PCR to identify the infected individual. When viral sequences were detected in an RNA-Seq or DNA-Seq dataset derived from a single toad, the sequence was amplified from sample cDNA or DNA if available. Australian toads, which were not subject to any NGS (*n*=64; Supplementary Table S1), were also screened for novel and known viruses using RT-PCR. Screening primers were designed based on contigs assembled from RNA-Seq or DNA-Seq data (Supplementary Table S1). cDNA was generated from cane toad RNA extracts using SuperScript™ VILO™ Master Mix (Thermo Fisher), and PCR was performed with *Taq* DNA Polymerase (NEB), unless otherwise specified. Amplicons generated were analyzed by agarose gel electrophoresis and Sanger sequenced at the Ramaciotti Centre for Genomics, UNSW, Sydney.

### Complete Genome Sequencing of Bufovirus A

We used RT-PCR to complete the unknown sequence gaps from a picornavirus detected in French Guianese toads. Liver cDNA from one individual (RMF033), who represented the only picornavirus positive toad in library RM_3, was used for all further sequencing. Partial picornavirus-like contigs (ranging in size from 203 to 815nt) from libraries RM_3 and RM_6 were first mapped against each other to confirm that they represented the same species of picornavirus (>99% nt identity).

The approximate sizes of the picornavirus genome gaps to be filled were determined by aligning known RM_3 contigs (*n*=5, ranging from 217 to 350nt) with closely related picornavirus genomes and designing primers to flank the sequence gaps. Primers used for amplification with Standard *Taq* Polymerase (NEB) and Sanger sequencing are listed in Supplementary Table S2.

Due to its inability to be amplified with Standard *Taq* Polymerase, a ~4kb gap spanning the VP2/2C region was amplified first by generating cDNA from RMF033 liver RNA using the SuperScript™ III First-Strand Synthesis System (Thermo Fisher) with tagged primer GV270 (5'-GCATGACTGACATAGCACAGCGGCCGCCCTTTTTTTTTTTTTTTTTTTTTTTTTTTTTT-3'), which anneals to the picornavirus poly(A) tail. The SequalPrep™ Long PCR Kit (Thermo Fisher) was then used to amplify the 4kb region flanked by primers MML212/MML205, consisting of 94°C denaturation for 2min, 10cycles of 94°C for 10s, 50°C for 30s, and 68°C for 6min, followed by 30cycles of 94°C for 10s, 50°C for 30s, and 68°C for 6min (+20s/cycle), and a final extension of 68°C for 5min. Samples were run on a 0.8% agarose gel and a band corresponding to the expected size was gel-extracted with the QIAquick® Gel Extraction Kit (Qiagen). A nested PCR was run on the gel extract under the same conditions using primers MML198/MML203, to generate a single amplicon of ~4kb. Additional inner primers (Supplementary Table S3) were then designed for further rounds of Sanger sequencing to span the whole amplicon.

Following generation of amplicons representing different regions of the picornavirus genome, Sanger sequencing reads and picornavirus-like Trinity contigs from RM_3 were assembled *de novo* in Geneious (v10.2). PCR and Sanger sequencing was repeated using different primer combinations until at least 2× coverage of the picornavirus polyprotein gene was obtained. Based on contigs generated from Sanger sequencing runs, the majority consensus (base called by >50% contigs) for each nucleotide position were chosen to produce the final polyprotein sequence.

To sequence the untranslated regions (UTRs) of the picornavirus genome, rapid amplification of cDNA ends (RACE) was performed. For the 3'-UTR previously published methods were followed (Katayama et al., 2002).

### Complete Sequencing of a Novel Papillomavirus Genome From Cane Toad Liver

Following the detection of papillomavirus-like contigs in two DNA-Seq datasets, we attempted to obtain additional papillomavirus sequence from DNA-Seq reads by extending Megahit contigs with GapFiller (v1.1.1; Nadalin et al., 2012) and then further scaffolding these contigs with SSPACE (v3.0; Boetzer et al., 2011). Primers were designed to amplify the unknown sequence between the lengthened contigs based on the typical papillomavirus gene order (Supplementary Table S2). Prior to PCR, DNA extracts from infected samples were enriched for circular DNA fragments using rolling circle amplification (RCA). RCA was performed in a 20μl reaction with 1.25U Phi29 DNA Polymerase (Thermo Fisher), 50μM exonuclease-resistant random hexamers (Thermo Fisher), 5μg BSA, 450μM dNTPs, and 2μl template DNA (<400ng). Reaction mixtures were incubated at 30°C for 18h followed by a 10min denaturation step at 65°C. RCA products were diluted 1/500 and used as PCR templates, with primers combined to amplify different portions of the circular genome. Lengthened contigs and Sanger sequencing reads were assembled *de novo* in Geneious (v10.2) to obtain the final circular papillomavirus genome sequence.

### Assessment of Sensitivity of Rhimavirus-A Screening Assay

Sixty-four Australian liver samples, which did not undergo RNA-Seq were screened for Rhimavirus-A (RhiV-A) *via* RT-PCR as previously described (Russo et al., 2018). The sensitivity of the PCR assay targeting the RhiV-A 5'-UTR was assessed by cloning the amplicon from a RhiV-A infected spleen sample into pGEM-T Easy with the pGEM-T Easy Vector System (Promega). To assess the lower detection limit of the target sequence, serial dilutions of the recombinant plasmid were used in a quantitative PCR using iTaq™ Universal SYBR® Green Supermix (Bio-Rad) with primers MML157/MML158 (Supplementary Table S2).

### Amplification and Sequencing of the RhiV-A VP1 Structural Region

In order to assess the diversity of the RhiV-A structural region, primers were designed to flank the sequence of the RhiV-A VP1 gene based on the type sequence (GenBank accession NC_040642). Partial or complete regions of the VP1 gene were then amplified by using forward primer MML248 (5'-GCTTATGATCCTGTGCCCGA-3') or MML247 (5'-GCCGTAGCACCTGAAGATGT-3') and reverse primer MML251 (5'-AGCTGGGGGTGTTTGTAGTG-3'), and Sanger sequenced as described.

### Phylogenetic Analysis of Novel Viral Sequences

To classify novel viruses in this study, representative viral sequences from known genera, or closely related isolates within the same viral family as the novel virus, were downloaded from NCBI. Regions used for phylogenetic analysis were chosen based on convention for that family. If only partial genome sequence was available for that virus, then all the available sequences were used. Protein alignments were performed with MAFFT (v7.407; Katoh et al., 2002) and trimmed with trimAL (v1.4.1; Capella-Gutierrez et al., 2009). Unrooted phylogenetic trees were inferred with RAxML (v8.2.12; Stamatakis, 2014) using a BLOSUM62 substitution matrix and 500 bootstrap replicates.

## Results

### Vertebrate-Infecting Viral Transcripts in Cane Toad Sequencing Data

To find viral transcripts using metatranscriptomics, eight pooled RNA-Seq libraries (RM_1–RM_8) were generated from 78 toads. Following assembly of these libraries and 19 additional publicly available RNA-Seq datasets, we discovered 65 vertebrate-like viral contigs resembling five viral families [*Iridoviridae* (*n*=47), *Picornaviridae* (*n*=15), *Hepeviridae* (*n*=1), *Nackednaviridae* (*n*=1) and *Caliciviridae* (*n*=1)].

To find DNA viral genomes we analyzed whole genome sequencing data from four French Guianese cane toads. Following the removal of host-aligned reads from DNA-Seq data, 10 vertebrate-specific viral sequences were identified from the *Papillomaviridae* (*n*=6) and *Circoviridae* (*n*=4).

### RhiV-A Infects Toads in All Ranges of Their Australian Habitat at a High Prevalence

**Figure 1:**
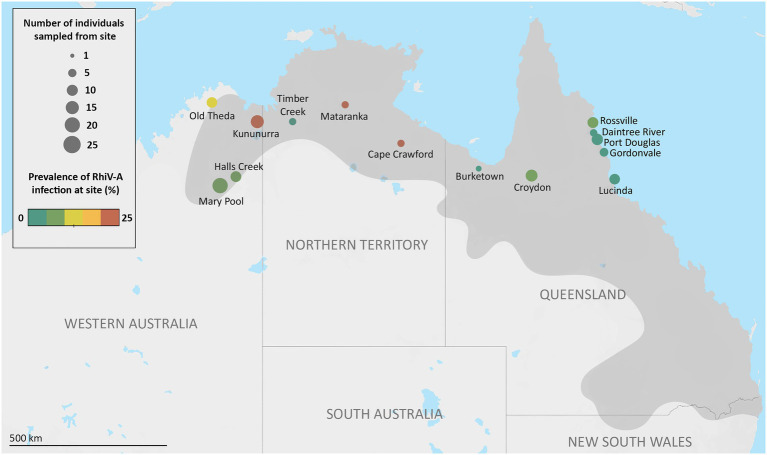
Individual sampling sites, and Rhimavirus-A (RhiV-A) prevalence across cane toads from Australia, 2015–2018. Number of sampled individuals from each site is indicated by the size of the circles, while prevalence of RhiV-A infection indicated by color scale. RhiV-A infection status was determined by performing RT-PCR targeting the RhiV-A 5' untranslated region from RNA extracted from either spleen or liver of sampled individuals. Darker gray coloring on the map represents an estimate of the cane toads’ current Australian distribution (Tingley et al., 2017).

Previously, we observed that the picornavirus RhiV-A actively infected cane toads from Australian locations over 1,000km apart (Russo et al., 2018). In the current study, we sought to assess the prevalence of RhiV-A across their range in northern Australia by screening additional cane toads (*n*=107) for infection *via* RNA-Seq and RT-PCR. Sampled toads were derived from all ends of their Australian range: their long-established QLD habitat (“range core”), geographically “intermediate” areas (NT), and the “invasion front” in WA where toads are rapidly advancing westwards (Figure 1).

Two pooled Australian transcriptomes (RM_1 and RM_7, 24 individuals total) contained contigs with identity to RhiV-A (89–99% aa identity). RT-PCR of RNA from toads comprising these pools revealed one toad with RhiV-A sequences in each library: from Mataranka, NT, and Mary Pool, WA, respectively (Figure 1). Screening a further 64 toads for the presence of RhiV-A using RT-PCR only demonstrated five more individuals from Rossville, QLD (*n*=1), Lucinda, QLD (*n*=1), Kununurra, WA (*n*=2), and Old Theda, WA (*n*=1; Figure 1). Both liver and spleen (depending on tissue availability) could be used to detect RhiV-A, demonstrating dual tissue tropism of the virus.

We combined these novel epidemiological data with our previous study to estimate RhiV-A’s geographical prevalence across AU. In total, RhiV-A infected 13 of 151 Australian toads analyzed (9%; Figure 1). RhiV-A infection spanned the toads’ entire Australian range, from the point of introduction (north-eastern QLD) to the current invasion front (close to Old Theda, WA; Figure 1). This updated RhiV-A range model encompasses 2,200km of latitudinal distance across northern AU (Figure 1). RhiV-A infection rates were almost five times higher in invasion front and intermediate areas (WA *n*=9/67, 13%, NT *n*=2/15, 13%) than in the range core (*n*=2/69, 3%; Figure 1). No RNA-Seq reads from any FG or HI dataset mapped to the RhiV-A genome.

### The RhiV-A VP1 Structural Region Exhibits Low Nucleotide Variation Across Australia

Relative to the highly conserved 5'-UTR commonly used for picornavirus diagnosis, the VP1 major capsid gene is more evolutionarily informative as it is subject to immune pressure (Thoelen et al., 2004). To assess the diversity of the RhiV-A VP1, we therefore amplified partial or complete VP1 sequences from nine RhiV-A infected toads from the following locations: Durack River and Kununurra WA (both *n*=2), Mataranka NT, Cape Crawford NT, Rossville QLD, Old Theda WA, and Halls Creek WA (all *n*=1; Figure 1). The VP1 region could not be amplified from three of 13 samples.

The nine RhiV-A VP1 sequences (610nt alignment) exhibited high nt identity to one another (93.3–100%). Moreover, sequence divergence broadly correlated with the geographic distance between the toads’ collection sites. Namely, two identical VP1 sequences (from RM0676S and RM0658S) were derived from cane toads in relative proximity (Durack River and Kununurra, WA, 190km apart). In contrast, the two most divergent isolates (93.3% nt identity) were from toads collected almost 2,000km apart (Rossville, QLD and Old Theda, WA).

### A Novel Picornavirus Infects Native Range Cane Toads

**Figure 2:**
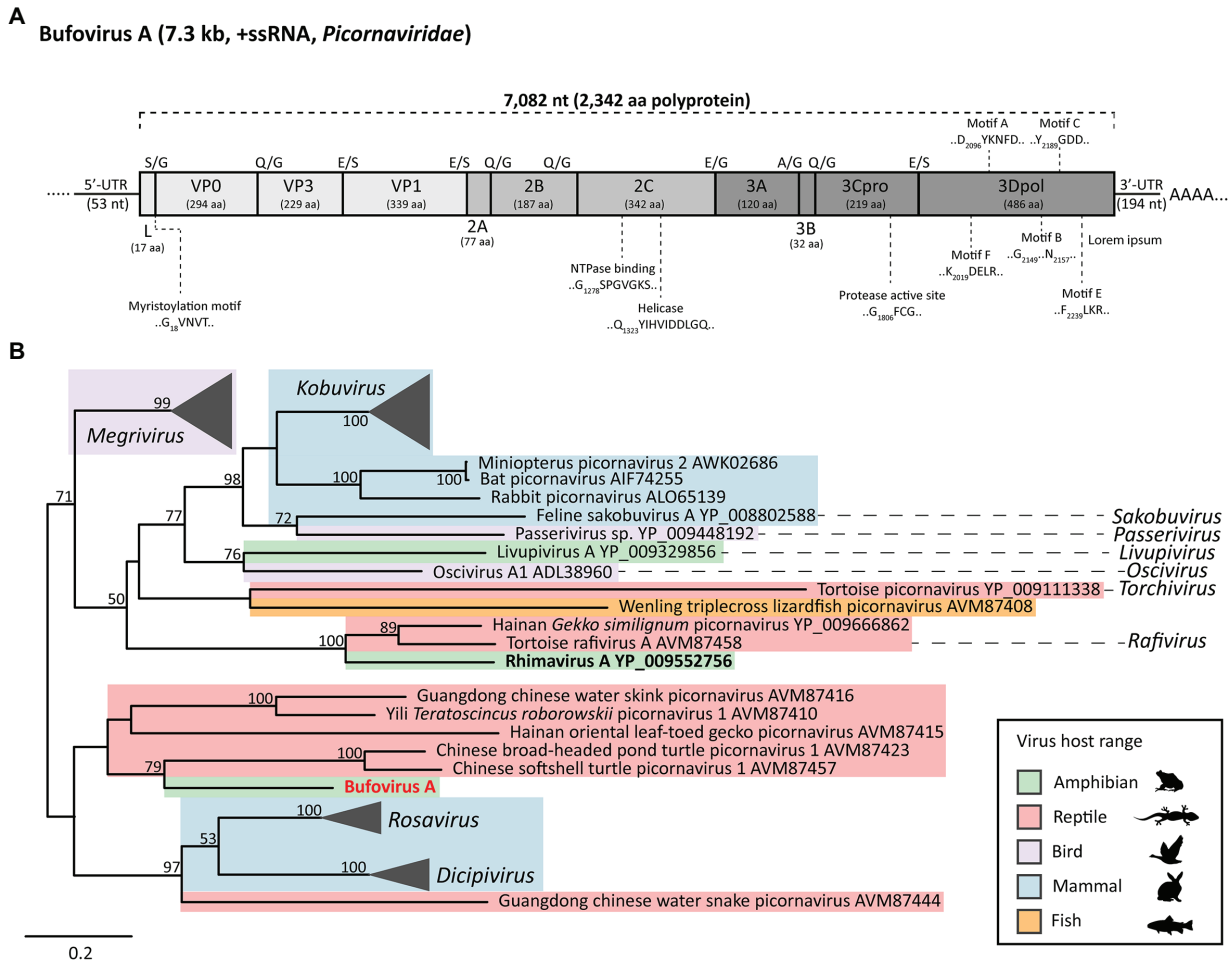
Genome structure and phylogenetic lineage of Bufovirus A (Buf-A) detected in two cane toads from French Guiana. **(A)** Genome organization of Buf-A. Predicted protease cleavage sites are indicated along the top of the polyprotein. Coloring indicates genomic region: light gray, P1, medium gray, P2, and dark gray, P3. Predicted size (amino acids) of each protein are indicated beneath the protein name. Conserved enzymatic motifs are indicated above or below the polyprotein. **(B)** Phylogenetic analysis of the Buf-A 3C_pro_/3D_pol_. Based on trimmed MAFFT alignment of the complete 3C/3D region of Buf-A1 and 51 other *Kobu*-like picornaviruses (484 aa positions). RAxML (v8.2.12) was used to generate a maximum likelihood phylogenetic tree with an LG substitution model and 500 bootstrap replicates. Shaded color represents host range. Scale bar represents aa substitutions per site. Bootstrap support (%) is indicated on each node; bootstrap values <50 are not shown.

We generated a single contig of 7,298nt encompassing a complete picornavirus polyprotein (7,082nt), 3'-UTR (194nt), and partial 5'-UTR (53nt; Figure 2A). The novel picornavirus was named Bufovirus A (Buf-A) after Bufonidae, the family containing true toads to which *R. marina* belongs. The Buf-A genome encodes a 2,342 aa polyprotein with typical picornavirus structure (L-VP0-VP3-VP1-2A-2B-2C-3A-3B-3C_pro_-3D_pol_; Figure 2A). Conserved enzymatic motifs were identified in the VP0, 2C, 3C_pro_, and 3D_pol_ regions (Figure 2A; te Velthuis, 2014). Notably, the myristoylation motif marking the VP0 N-terminus occurred after the polyprotein start codon, meaning Buf-A has an unusually small L protein, if cleaved (17 aa, this ranges from 52 to 462 aa in other picornaviruses). According to a phylogeny of the 3C/3D region, Buf-A forms a distinct clade with gecko and turtle viruses (Figure 2B). Buf-A is also phylogenetically distinct from RhiV-A; however, they both can be classified among the “*Kobuvirus*”-like genera (Figure 2B). RT-PCR screening revealed that 2/25 of sampled French Guianese toads were infected with Buf-A. No reads mapping to Buf-A were identified in any RNA-Seq dataset from AU or HI, and all *n*=64 Australian toads screened *via* RT-PCR for Buf-A were negative (data not shown).

### A Highly Divergent Papillomavirus Infects French Guianese Cane Toads

**Figure 3:**
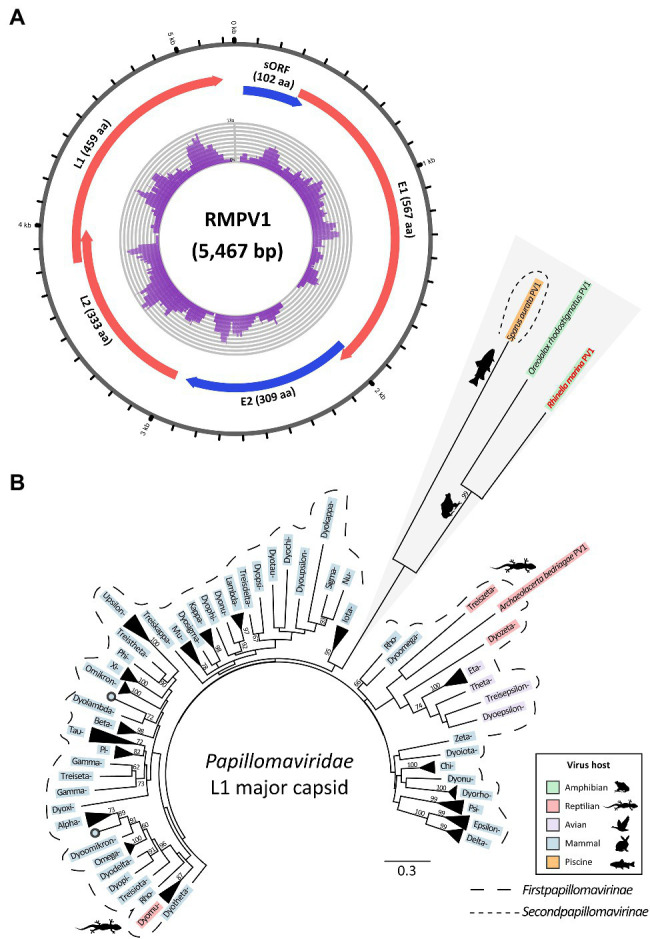
Genome and lineage of a novel papillomavirus (*Rhinella marina* papillomavirus 1, RMPV1) detected in three cane toads in French Guiana. **(A)** Genome organization of RMPV1. Numbering around the genome refers to nucleotide position (kb). Predicted open reading frames (ORFs), which correspond to conserved papillomavirus proteins are indicated in red or blue; those in the same color are encoded in the same ORF. Purple histogram in the center denotes DNA-Seq read coverage from individual RMF042. Sequence not represented by DNA-Seq reads was determined with rolling circle amplification-enriched polymerase chain reaction. Image created with Circos (Krzywinski et al., 2009). **(B)** L1 major capsid phylogeny of RMPV1 and other papillomaviruses. Based on MAFFT alignment of the RMPV1 L1 protein coding region and 83 other papillomaviruses (284 aa positions). RAxML (v8.2.12) was used to generate a maximum likelihood phylogenetic tree (LG substitution matrix with 500 bootstrap replicates). Within the *Firstpapillomavirinae* subfamily (longer dotted line), official genera are noted by their Greek letter prefix, e.g., *Dyokappa=Dyokappapapillomavirus*. Scale bar represents aa substitutions per site. Bootstrap support (%) is indicated next to nodes; values <50 are not shown.

To find DNA virus genomes, we also analyzed total DNA-Seq data from the livers of four French Guianese cane toads. Contigs with similarity to papillomavirus protein sequences (323–1,732nt) were present in three of four DNA assemblies. The sample generating the longest of these contigs (toad RMF042) was used to complete the novel genome (5,467bp); this virus was denoted *R. marina papillomavirus* 1 (RMPV1, Figure 3A).

Papillomavirus-like contigs from the two additional infected toads (RMF044 and RMF048) exhibited 99.5–99.9% identity to the RMPV1 genome, indicating that all three toads were infected with the same viral species. Raw reads from infected DNA-Seq datasets, and available RNA-Seq datasets, were also mapped to the RMPV1 genome to assess relative viral tissue abundance and transcription. RMPV1-specific raw DNA reads were sparse, varying from 71 to 182 reads per sample. No RNA-Seq reads from pools containing infected individuals mapped to the RMPV1 genome, indicating undetectable viral transcription.

*Rhinella marina papillomavirus* 1 is related to two other papillomaviruses infecting toad and fish: *Oreolalax rhodostigmatus* (Guizhou lazy toad) PV1 (OrPV1) and *Sparus aurata* (gilt-head bream) papillomavirus 1 (SaPV1; NC_030839; Figure 3B). These form distinct lineages from first papillomaviruses (Figure 3B), indicating a huge unsampled pool of papillomaviruses in cold-blooded hosts perhaps comprising additional members of the *Secondpapillomavirinae*. RMPV1 shares genomic characteristics with SaPV1, the only member of the *Secondpapillomavirinae* with a fully sequenced genome. Both viruses exhibit reduced genomes (5.5–5.7kb) compared to the *Firstpapillomavirinae* subfamily (~8kb) yet maintain typical papillomavirus genome structure with the four core genes in the order E1-E2-L2-L1 (Figure 3A). The RMPV1 long control region (160nt) is comparatively small compared to other papillomaviruses (~850nt) but still contains conserved regulatory elements including an AT-rich region (24nt) and a 22nt palindromic sequence (5'-TATTATTGTGTACACAATAATA-3'). Instead of E6 and/or E7 genes, RMPV1 contained one putative small ORF of 102 aa, which lacked significant BLAST database matches (Figure 3A).

### A Novel Erythrocytic-Like Iridovirus Infects French Guianese Cane Toads

**Figure 4:**
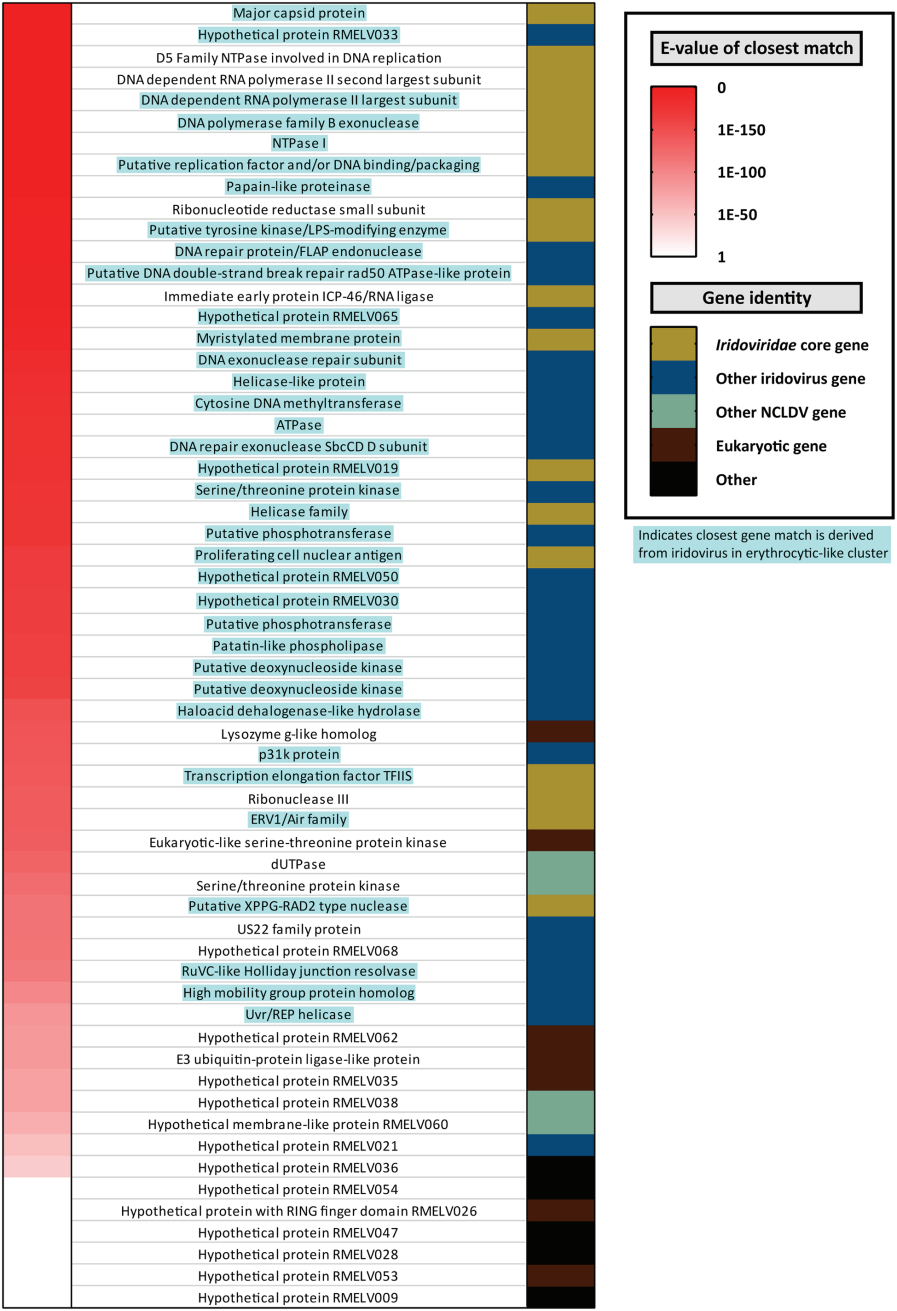
Annotation of putative proteins encoded by a novel iridovirus detected in French Guianese cane toads, *Rhinella marina* erythrocytic-like iridovirus (RMELV). Seventy-two putative protein coding ORFs were identified in a pooled liver transcriptome (RM_6) from 10 French Guianese cane toads. Left hand column indicates Expect value (*E*-value) of the RMELV ORFs top hits when queried against the NCBI *nr* database, with red indicating a closely related match (*E*-value approaching zero) and white indicating a more distantly related match (*E*-value approaching one). Right hand column indicates the type of ORF encoded by the virus. Gold indicates core *Iridoviridae* genes (Eaton et al., 2007). Dark blue indicates close matches to another iridovirus protein. Light green indicates a match to a protein from other nucleocytoplasmic large DNA viruses. Maroon indicates matches to eukaryotic genes, and black indicates an ORF that lacks a match in the *nr* database. In the middle, ORFs are labeled by the putative protein, which they encode as determined by BLASTp search, with attempts to conserve gene nomenclature in the *Iridoviridae*. Blue highlighted gene names indicate that the closest match is from a virus within the erythrocytic-like iridovirus cluster.

**Figure 5:**
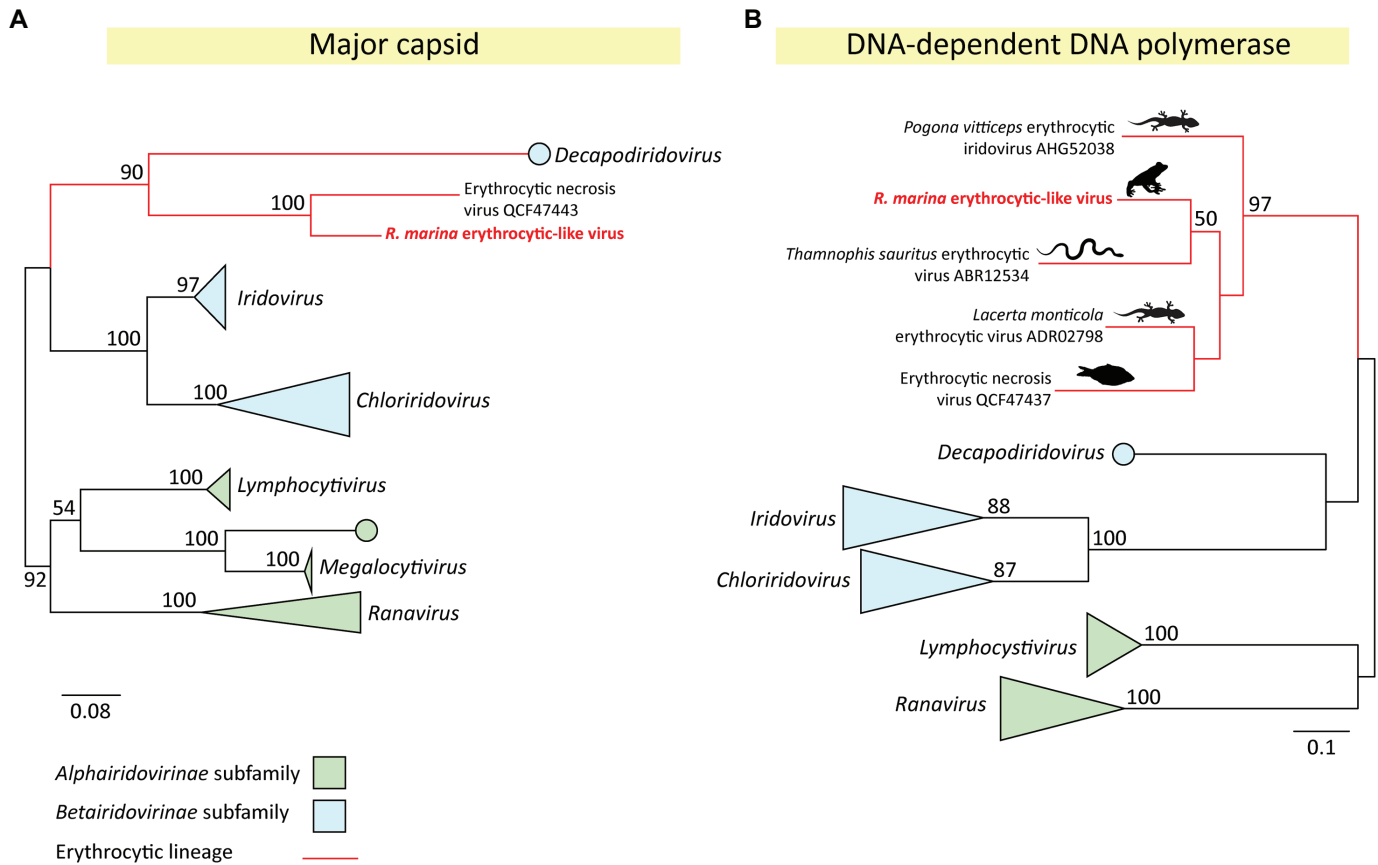
Phylogeny of a novel iridovirus (*Rhinella marina* erythrocytic-like virus, RMELV) from toads in French Guiana. **(A)** Maximum likelihood (ML) phylogeny of the major capsid protein (MCP) gene (314 aa positions) of RMELV and 35 other iridoviruses. **(B)** ML phylogeny of the DNA-dependent DNA polymerase (DNA family B exonuclease) protein (172 aa positions) of RMELV and 39 other iridoviruses. Alignments created with MAFFT (v7.407), trimmed with trimAL (v1.4.1), and phylogenies inferred with RAxML (v8.2.12) using an LG **(A)** or WAG **(B)** aa substitution model. Official genera are italicized. Lineages classified within the *Alphairidovirinae* are indicated in green, and *Betairidovirinae* in blue, with strongly supported clades collapsed. The erythrocytic lineage is shown with red branches; images indicate host type. Node labels indicate bootstrap support from 500 replicates, support values <50% are not shown. Scale bar represents aa substitutions per site.

The erythrocytic-like clade of the *Iridoviridae* can be highly pathogenic in reptiles and fish. Herein, we identified a cane toad-infecting member of this group and characterized its near-complete transcriptome. The novel virus was denoted *R. marina* erythrocytic-like virus (RMELV) and infected the liver of 2/24 sampled French Guianese cane toads. Based on major capsid phylogeny, RMELV’s closest known relative is the fish-infecting ENV (75% aa identity; Figures 4, 5).

The RMELV transcriptome comprised 39 contigs totaling 80,888nt viral mRNA. This mRNA encoded 72 putative ORFs (20,931 aa total), half of which (*n*=36) generated significant matches to erythrocytic iridovirus proteins (Figure 4; Supplementary Table S4). A further eight RMELV ORFs matched proteins from other *Iridoviridae* genera; together with the erythrocytic-like ORFs, all 26 core *Iridoviridae* family proteins as defined by Eaton et al. (2007) were present (Figure 4). Four additional ORFs exhibited identity to proteins from other nucleocytoplasmic large DNA viruses (NCLDVs; Supplementary Table S4). ORFs encoded on an RMELV contig, but with no iridovirus match, were also classified as viral in origin (*n*=24). Of these 24 ORFs, seven generated matches to eukaryotic proteins of which three were assigned putative functions (Figure 4; Supplementary Table S4). A further 17 ORFs with no viral/eukaryotic match were denoted “hypothetical proteins” (Figure 4; Supplementary Table S4). No reads from any other RNA-Seq or DNA-Seq dataset mapped to RMELV contigs. None of the additional 64 Australian toads tested positive for RMELV by RT-PCR.

### RMELV Is a Betairidovirus Distinct From All Other Known Amphibian Iridoviruses

To further classify the novel cane toad iridovirus, we inferred a maximum likelihood (ML) capsid phylogeny of RMELV and 35 other iridoviruses representing all described genera. This confirmed our observation that RMELV and ENV are close relatives; the two viruses clustered together (Figure 5A) and share high capsid similarity (75% aa). Together, they form a clade which clusters with the *Betairidovirinae* subfamily members. Although betairidoviruses were traditionally thought to be an invertebrate-dominated group, our findings further support the existence of betairidovirus-like viral clades infecting vertebrates (Pagowski et al., 2019).

In addition to the capsid protein, the iridovirus DNA-dependent DNA polymerase (DdDp) is a reliable phylogenetic marker and is often used as a PCR target. Notably, the RMELV DdDp sequence generated BLAST hits to three reptile viruses lacking any additional sequence information, suggesting further diversity in the erythrocytic clade. These were *Lacerta monticola* (Iberian rock lizard) erythrocytic virus (72% identity over 199 aa; de Matos et al., 2011), *Thamnophis sauritus* (ribbon snake) erythrocytic virus (70% over 210 aa; Wellehan et al., 2008), and *Pogona vitticeps* (central bearded dragon) erythrocytic virus (64% identity over 146 aa; Grosset et al., 2014). To further investigate RMELV’s relationship to these viruses, we inferred a DdDp phylogeny to show that these pathogenic reptile viruses also belong in this erythrocytic betairidovirus clade along with ENV and RMELV (Figure 5B). Altogether, we describe perhaps the first erythrocytic amphibian iridovirus and indicate untapped viral diversity in the cluster.

### Partial Amphibian- and Fish Virus-Like Sequences Indicate Wider Viral Diversity in Cane Toads From Three Countries

Viruses of amphibians and bony/cartilaginous fish are often closely related, corresponding to the evolution of their vertebrate hosts (Shi et al., 2018). Based on this criterion, we identified two partial viral genomes in our data which we deduced to also be from cane toad-infecting viruses, a nackednavirus and a hepevirus.

“Nackednaviruses” are proposed non-enveloped ancestral hepadnaviruses (Lauber et al., 2017). In an RNA-Seq dataset from French Guianese spleen sample RMF048S, a 356nt contig generated a BLASTx hit to the polymerase gene of African cichlid nackednavirus (MH158727; *E*-value 6.02e^−23^, 52% aa identity over 91 aa) and clustered with two other nackednavirus-like sequences (Supplementary Figure S1A). Many new hepatitis E-like (“hepevirus-like”) viruses have recently been found in fish. A 202nt sequence in Australian pooled transcriptome RM_8 generated a BLAST hit to the capsid region of Nanhai ghost shark hepevirus (MG600008; *E*-value 6.11e^−22^, 70% identity over 66 aa). These clustered phylogenetically with other hepevirus capsid sequences from ray-finned fish (Supplementary Figure S1B). More distant matches were to astroviruses, according with the common capsid ancestry of hepeviruses and astroviruses (Kelly et al., 2016). We PCR amplified the two above sequences from cDNA of infected toads but were unable to generate any additional genome sequence.

To further investigate the cane toad virome across countries not encompassed by our collection, we examined a liver RNA-Seq dataset from a Danish captive cane toad. Herein, we found a 258nt calicivirus-like RdRp sequence generating a match to Fujian spotted paddle-tail newt calicivirus (*E*-value 2.61e^−15^, 47% identity over 86 aa). The sequence clustered with other caliciviruses from fish, amphibians, and reptiles (Supplementary Figure S1C), supporting its host specificity. The sequences indicate wider viral diversity in cane toads from many countries, as well as under-sampled fish and amphibian viral clades.

### Diverse Non-vertebrate-Infecting Viral Transcripts Are Detectable in Cane Toad Metatranscriptomic Data

Animal metatranscriptomics may uncover viruses infecting host symbionts such as endo- and ecto-parasites (Mahar et al., 2020). In our RNA-Seq libraries, we found 58 transcripts related to viruses infecting non-vertebrate eukaryotes including arthropods, fungi, protozoa, molluscs, and plants (Supplementary Table S5); these viruses were likely infecting a non-vertebrate cane toad symbiont. The viral groups of origin were hugely diverse and included +ssRNA viruses (*Dicistroviridae*, *Polycipiviridae*, and *Narna-*like clade), segmented dsRNA viruses (*Partiti-*like clade), non-segmented dsRNA viruses (*Endornaviridae*, *Toti*-like clade), segmented −ssRNA viruses (*Orthomyxoviridae* and *Bunyavirales*) and non-segmented −ssRNA viruses (*Mononegavirales* and *Chuviridae*; Supplementary Table S5).

Non-vertebrate RNA viruses from French Guianese cane toads were both more numerous (55 transcripts) and more diverse (10 distinct viral groups) than in Australian toads (*n*=3 transcripts from one viral group). Further, all three French Guianese RNA-Seq libraries contained non-vertebrate viruses, compared to one of five Australian libraries (Supplementary Table S5).

Viral transcripts of the same family and toad pool were classified as originating from the same virus and were named according to their viral family: e.g., Cane toad-associated orthomyxovirus. In several cases, the sequence obtained amounted to a substantial portion of the expected viral genome size: we identified approximately 90% of a bunyavirus genome (Cane toad-associated bunyavirus, 6.6/~7.5kb) and ~95% of a totivirus-like genome (Cane toad-associated toti-like virus 3, 6.7kb/7kb; Supplementary Table S5). Viral sequences from the same families, but in different RNA-Seq pools, shared no homology, suggesting distinct viral genomes in each sample. Overall, native-range toads contained a richer abundance of non-vertebrate-associated viruses than did introduced-range toads.

## Discussion

For the last 80years, poisonous cane toads have spread through Australia at an accelerating rate (Phillips et al., 2006). Continued range expansion into diverse habitats is predicted and is likely to exacerbate the toads’ impact on native fauna (Murray and Hose, 2005). Therefore, we need to understand and hopefully mitigate the cane toads’ ongoing invasion. Finding viruses is one way to do this, as viruses may have drastic effects when brought by an invasive species. Additionally, viruses may be used as successful biological control tools for invasive species. These are strong justifications for finding novel viruses in cane toads.

We previously described three viral families associated with cane toads. However, the full spectrum of cane toad viruses is likely to extend beyond these three families, and comparisons between introduced and native viromes are lacking. In this study, we performed a multi-continental, deep-sequencing survey of the cane toad metavirome in Australia (an introduced range) and South America (the toad’s native range) to clarify the interplay between viruses and the invasion process. We additionally sought to investigate the toad’s ancestral virome to find potential biological controls.

### RhiV-A Is Geographically Ubiquitous Across the Australian Cane Toad Range

We previously characterized RhiV-A, a kobu-like picornavirus that was likely to be infecting Australian cane toads (Russo et al., 2018). In the current study, we demonstrated that the virus is geographically ubiquitous, detectable in cane toads from the range core (coastal QLD), intermediate areas (NT/inland QLD), and invasion front (WA) spanning approximately 2,000km of latitudinal distance (Figure 1). This suggests that RhiV-A was likely present in the founder population (or acquired from another species very soon after arrival), accompanying the toads as they spread westwards across the continent.

### Australian Cane Toads Have High Rates of RhiV-A Infection

As well as occurring across the toads’ entire Australian range, RhiV-A was highly prevalent with a 9% infection rate (Figure 1). Such a high rate is rare in natural host populations, where picornaviruses usually cause acute and transient infections. High rates of picornavirus infection (~10% or above) are often the result of sporadic outbreaks in a dense host population (e.g., farm animals; Boros et al., 2020). These conditions were not present in our study, as infected toads had low host density, were geographically separated, and were collected over 4years. Therefore, if RhiV-A has an acute pattern of infection like most picornaviruses, other factors are required to maintain its prevalence. For example, infection with RhiV-A may not protect against re-infection.

An alternative explanation for high RhiV-A rates is chronic (long-term) infection of cane toads. In other animals, chronic picornavirus infection only occurs in a minority of individuals who are often immunocompromised (Randall and Griffin, 2017). Therefore, chronic infection would also suggest some immune deficiency of cane toads, stopping them from clearing the infection. Interestingly, a lack of immune pressure could be reflected in RhiV-A’s structural homogeneity (<7% VP1 nt divergence). RhiV-A’s infection pattern clearly warrants further study; but immune deficiency among cane toads is one possible explanation for the virus’s homogeneity and prevalence. This could reflect low investment in immune defenses by range-edge cane toads, so that they clear RhiV-A infection less readily than do conspecifics in long-colonized areas (Llewellyn et al., 2012).

### Buf-A, a Native Range Cane Toad Picornavirus, Enriches Amphibian-Specific Lineages in the *Picornaviridae*

We next examined the cane toad’s native virome by sequencing South American cane toad viruses. The native range may harbor potential biological control viruses, as invasive range toads will likely lack immunological exposure to them. We found a distinct picornavirus in South America, Buf-A, present in liver of two of 24 sampled French Guianese cane toads (Figure 2).

Both Buf-A and RhiV-A are *Kobu*-like picornaviruses, but they are genetically distinct (Figure 2B). This indicates that cane toads harbor multiple *Kobu*-like picornavirus lineages. However, fewer than 10 picornaviruses infecting amphibians have been sequenced, indicating potentially thousands of unresolved viruses among the *Kobu* clade (Reuter et al., 2015; Pankovics et al., 2017; Shi et al., 2018). The identification of both Buf-A and RhiV-A helps to expand knowledge of the *Picornaviridae*, which will facilitate future amphibian picornavirus studies.

### RMPV1 Exemplifies Ancestral Gene Structure Within the *Papillomaviridae*

Amphibians and fish are largely absent as hosts from the *Papillomaviridae*, with the only fully sequenced member (SaPV1 – infecting bream) placed within its own subfamily, the *Secondpapillomavirinae* (Van Doorslaer et al., 2018). Herein, we demonstrated additional diversity of amphibian and fish papillomaviruses by sequencing a cane toad papillomavirus, RMPV1. RMPV1 forms a well-supported monophyletic lineage with *O. rhodostigmatus* papillomavirus 1, but not with other papillomaviruses, suggesting that together these two viruses may be classified in an additional third subfamily (Figure 3B). Genetic distance among these viruses exceeds that of all *Firstpapillomavirinae* lineages further supporting the possibility of multiple subfamilies (Figure 3B). Like SaPV1, the RMPV1 genome (Figure 3A) resembles the ancestral “proto-papillomavirus” (URR-E1-E2-L2-L1; Garcia-Vallve et al., 2005) lacking the E6/E7 transforming genes, which were hypothesized to have arisen following vertebrate diversification ~500 million years ago. Despite their shared lineage, RMPV1 and SaPV1 share low aa similarity (≤23%), suggesting special demarcation criteria are needed for these divergent papillomaviruses. Altogether, our results suggest that there is a huge untapped papillomavirus pool in fish and amphibians, with each animal potentially hosting as many papillomaviruses as humans do (>100; Van Doorslaer et al., 2018). Its presence in Australian toads represents an important future avenue of investigation.

### RMELV Is a Novel Species in the Highly Pathogenic Erythrocytic-Like Iridovirus Clade

Iridoviruses are large dsDNA viruses infecting fish, amphibians, reptiles, and invertebrates (Chinchar et al., 2017). In the 1990s, six iridovirus isolates from Venezuelan cane toads were trialed as biological control agents against Australian toads and exhibited some promise (Hyatt et al., 1998; Shannon and Bayliss, 2008); however, they were not sequenced. Herein, we accurately classify a South American cane toad iridovirus, RMELV.

*Rhinella marina* erythrocytic-like virus clustered into a well-supported “erythrocytic-like” iridovirus clade (Figure 5) that is distinct from the genus *Ranavirus* – previously thought to be the only amphibian-infecting lineage. Erythrocytic-like iridoviruses cause severe blood disorders in reptiles and fish, resulting in anorexia, anemia, dehydration, necrotizing hepatitis, and death (Johnsrude et al., 1997; Wellehan et al., 2008; Hershberger et al., 2009; Grosset et al., 2014). Isolation of RMELV and challenging immunologically naïve toads will help to clarify its efficacy as a candidate for biological control.

Interestingly, the erythrocytic-like cluster is more closely related to the *Betairidovirinae* subfamily, thought to contain mostly viruses of invertebrates, than to the *Alphairidovirinae*, which is thought to harbor most vertebrate-infecting iridoviruses (Figure 5). The demarcation of erythrocytic-like viruses into their own genus within the *Betairidovirinae* may facilitate future classification of related viruses. Clearly, the *Iridoviridae* remains under-sampled, and the two subfamilies have evolved to independently infect the same class of host.

### Partial Viral Transcripts Indicate Richer Viral Diversity in Cane Toads From Australia, Europe, and South America

We identified three further putative viral transcripts from the *Nackednaviridae* (French Guianese toad), *Caliciviridae* (Danish laboratory toad), and *Hepeviridae* (Australian toad) in our RNA-Seq datasets (Supplementary Figure S1). These transcripts were likely to be derived from cane toad specific viruses as they clustered phylogenetically with fish viruses, which commonly form sister clades to amphibian viruses (Supplementary Figure S1; Shi et al., 2018). Evidently, the full extent of viruses infecting cane toads across all continents requires further research to fully understand the impact of viruses on toads throughout varied geographical regions and environmental conditions.

### Virome Structures Between Native and Introduced Range Toads May Be Shaped by the Invasion Process

Above, we hypothesized that RhiV-A was present in the cane toad founder population and then spread across the continent resulting in its current Australia-wide distribution (Figure 1). This implies that RhiV-A was once present in South America and/or HI, accompanying the toads throughout their translocations; however, it could have also been acquired from a native Australian animal. Additional sampling of international toads is required to confirm the origin of RhiV-A, as well as sampling other related Australian species for infection.

We also noted that the diversity of viruses is different between invasive and native cane toad populations. Namely, we detected two viral families in Australia (*Picornaviridae* and *Hepeviridae*) from 151 individuals, compared to four viral families from 25 individuals in FG (*Iridoviridae*, *Papillomaviridae*, “Nackednaviridae,” and *Picornaviridae*). Although our study lacked sufficient sampling size to ensure statistical rigor, future studies should employ extensive sampling of cane toads to test for the viruses found in this study.

### Cane Toads Harbor a Diverse Non-vertebrate Metavirome

Metatranscriptomic studies have recently identified thousands of novel viral genomes from invertebrate samples, which are usually phylogenetically distinct from vertebrate viral families (Li et al., 2015a; Shi et al., 2016).

In four of our pooled liver datasets (RM_3, RM_5, RM_6, and RM_8), we detected 58 transcripts related to invertebrate-, fungus-, or protozoan-specific RNA viral genomes; these numerous viral sequences likely reflect viral infections of cane toad symbionts or from their food sources (Supplementary Table S5). This is no surprise, as cane toads have an eclectic diet (Freeland et al., 1986) and harbor diverse eukaryotic fauna including protozoans, metazoans, and arthropods (Selechnik et al., 2017), many of which infect the liver from where the bulk of this paper’s data was derived. Due to the high load of visceral parasites often infecting cane toads, the most logical conclusion is that the host of these putative non-vertebrate infecting viruses are parasites. We observed that the French Guianese toad metaviromes contained substantially more invertebrate-associated viruses than did Australian toads (FG *n*=13, AU *n*=1; Supplementary Table S5), which may provide some further evidence for viral enemy release.

Although the viral groups described here usually exclusively infect non-vertebrate hosts, it is worth noting that recent literature has identified several instances of unexpected dual host range among such families. For example, totiviruses may infect both arthropods and fish (Haugland et al., 2011), while a recent paper described circulation of a marine flavivirus between vertebrate and non-vertebrate hosts (Parry and Asgari, 2019). Therefore, to determine the definitive hosts of these viruses further experimentation is needed.

## Conclusion

This study provides novel insights into the virome of the cane toad both in its native range and in its introduced range. We sequence novel viruses in the native range of the invasive cane toad, which appear to be absent in the Australian range and so which may be good candidates for toad biological control. Toads should be screened for incidence of these viruses in future studies to further assess their prevalence. Importantly, the host range of viruses in this study should be definitively established, which are only presumptive here. It is possible that the toads sampled in this study also possess divergent viruses, which were not detected with the methods used here, and therefore additional means of viral detection could be considered in future studies. Lastly, the increase in described viruses helps to expand the vastly under-sampled amphibian virosphere.

## Data Availability Statement

RNA-Seq data generated in this study are deposited in the SRA under BioProject accession PRJNA662839. DNA-Seq data generated in this study are available upon request. Viral sequences in this study are deposited under GenBank accessions MW582899–MW583016.

## Ethics Statement

All procedures involving live animals were approved by the University of Sydney Animal Care and Ethics Committee (2014/562), the Deakin University Animal Ethics Committee (AEX04-2014), and the University of Adelaide Animal Ethics Committee (S-2018-056).

## Author Contributions

AR and PW designed the research. AR, SD, JD, JZ, RS, MR, and LR conducted the fieldwork. AR, GY, EH, DS, YL, MR, and LR conducted the lab work. AR, EH, and PW conducted the data analyses. AR and PW drafted the manuscript. All authors contributed to the article and approved the submitted version.

## Conflict of Interest

The authors declare that the research was conducted in the absence of any commercial or financial relationships that could be construed as a potential conflict of interest.

## Publisher’s Note

All claims expressed in this article are solely those of the authors and do not necessarily represent those of their affiliated organizations, or those of the publisher, the editors and the reviewers. Any product that may be evaluated in this article, or claim that may be made by its manufacturer, is not guaranteed or endorsed by the publisher.
