# Supplementary material for: Discovery of Novel Viruses Associated With the Invasive Cane Toad (Rhinella marina) in Its Native and Introduced Ranges
Source: Front Microbiol. 2021 Sep 6;12:733631. doi: 10.3389/fmicb.2021.733631 (PMC8450580; doi:10.3389/fmicb.2021.733631)
Supplement: Supplementary file 1 [file Data_Sheet_1.docx]

Supplementary Material


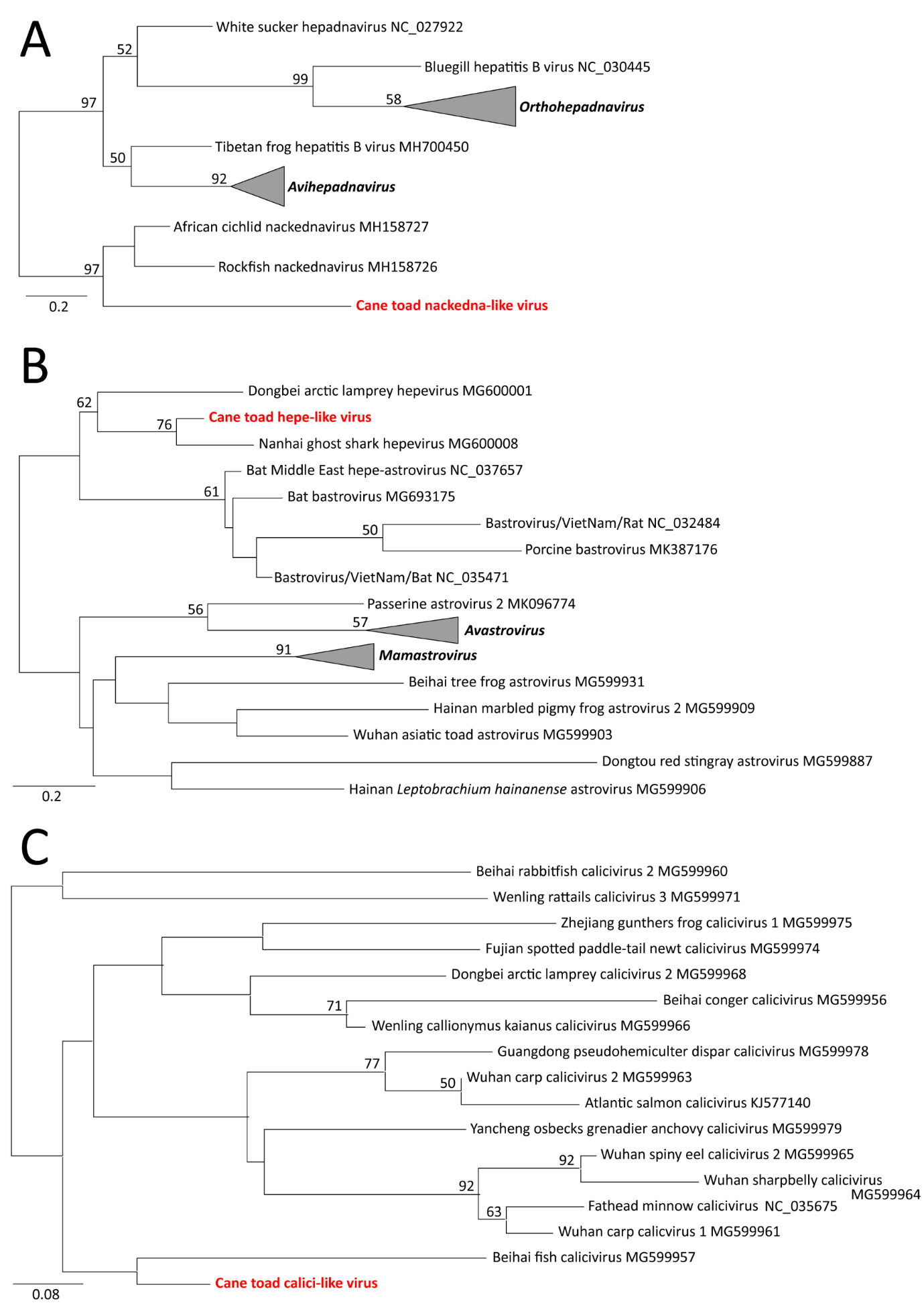


**Figure S1**. **Phylogenies of three partial viral genomes from cane toad metatranscriptomic data with other closely related vertebrate viruses**. **A)** Phylogeny of a nackednavirus DNA polymerase-like transcript in splenic transcriptome from French Guyanese individual RMF048. **B)** Hepevirus capsid-like transcript from individual RMLV17 from Port Douglas, QLD, AU**. C)** Calicivirus RdRp-like transcript from a liver transcriptome from a cane toad from a commercial supplier in Denmark. Closely related viral sequences of the corresponding regions as determined by BLAST were aligned with MAFFT (v7.407), trimmed with trimAL (v1.4.1), and phylogenies were inferred with RAxML (v8.2.12) using an LG aa substitution model. Length of alignments are 97 (A) 40 (B) or 50 (C) aa positions. Official ICTV genera are indicated in bold italic type. Node labels indicate bootstrap support (%) from 500 replicates; support values <50 are not shown. Viral sequences described in this study are shown in red. Scale bar represents aa substitutions per site.

**Table S1: Details of cane toad samples analyzed in this study.**

| **AUSTRALIA** | | | | | | | | |
| --- | --- | --- | --- | --- | --- | --- | --- | --- |
| Sample ID | Tissue type | Collection location | Collection state | Country of origin | Sex | Date of collection | Type of sequencing performed | RNA-Seq library number |
| RM0568 | Spleen | Mataranka | Northern Territory | Australia | M | Apr-15 | Inverse rRNA RNA-Seq (pooled) | 1 |
| RM0569 | Spleen | Mataranka | Northern Territory | Australia | M | Apr-15 | Inverse rRNA RNA-Seq (pooled) | 1 |
| RM0255 | Spleen | Gordonvale | Queensland | Australia | M | May-14 | Inverse rRNA RNA-Seq (pooled) | 1 |
| RM0293 | Spleen | Gordonvale | Queensland | Australia | M | May-14 | Inverse rRNA RNA-Seq (pooled) | 1 |
| RM0306 | Spleen | Croydon | Queensland | Australia | M | May-14 | Inverse rRNA RNA-Seq (pooled) | 1 |
| RM0502 | Spleen | Mataranka | Northern Territory | Australia | M | Apr-15 | Inverse rRNA RNA-Seq (pooled) | 1 |
| RM0516 | Spleen | Mataranka | Northern Territory | Australia | M | Apr-15 | Inverse rRNA RNA-Seq (pooled) | 1 |
| RM0518 | Spleen | Cape Crawford | Northern Territory | Australia | M | Apr-15 | Inverse rRNA RNA-Seq (pooled) | 1 |
| RM0575 | Spleen | Mataranka | Northern Territory | Australia | M | Apr-15 | Inverse rRNA RNA-Seq (pooled) | 1 |
| RM0599 | Spleen | Timber Creek | Northern Territory | Australia | M | May-15 | Inverse rRNA RNA-Seq (pooled) | 1 |
| RM0625 | Spleen | Caroline Pool | Western Australia | Australia | M | May-15 | Inverse rRNA RNA-Seq (pooled) | 1 |
| RM0695 | Spleen | Durack River | Western Australia | Australia | M | May-15 | Inverse rRNA RNA-Seq (pooled) | 1 |
| RM0313 | Spleen | Croydon | Queensland | Australia | F | May-14 | Inverse rRNA RNA-Seq (pooled) | 4 |
| RM0353 | Spleen | Burketown | Queensland | Australia | F | May-14 | Inverse rRNA RNA-Seq (pooled) | 4 |
| RM0384 | Spleen | Burketown | Queensland | Australia | F | May-14 | Inverse rRNA RNA-Seq (pooled) | 4 |
| RM0398 | Spleen | Burketown | Queensland | Australia | F | May-14 | Inverse rRNA RNA-Seq (pooled) | 4 |
| RM0621 | Spleen | Caroline Pool | Western Australia | Australia | F | May-15 | Inverse rRNA RNA-Seq (pooled) | 4 |
| RM0623 | Spleen | Caroline Pool | Western Australia | Australia | F | May-15 | Inverse rRNA RNA-Seq (pooled) | 4 |
| RM0655 | Spleen | Caroline Pool | Western Australia | Australia | M | May-15 | Inverse rRNA RNA-Seq (pooled) | 4 |
| RMLV1 | Liver | Mary Pool | Western Australia | Australia | F | Aug-18 | Inverse rRNA RNA-Seq (pooled) | 7 |
| RMLV2 | Liver | Mary Pool | Western Australia | Australia | M | Aug-18 | Inverse rRNA RNA-Seq (pooled) | 7 |
| RMLV3 | Liver | Mary Pool | Western Australia | Australia | M | Aug-18 | Inverse rRNA RNA-Seq (pooled) | 7 |
| RMLV4 | Liver | Mary Pool | Western Australia | Australia | M | Aug-18 | Inverse rRNA RNA-Seq (pooled) | 7 |
| RMLV5 | Liver | Mary Pool | Western Australia | Australia | M | Aug-18 | Inverse rRNA RNA-Seq (pooled) | 7 |
| RMLV6 | Liver | Mary Pool | Western Australia | Australia | M | Aug-18 | Inverse rRNA RNA-Seq (pooled) | 7 |
| RMLV7 | Liver | Mary Pool | Western Australia | Australia | M | Aug-18 | Inverse rRNA RNA-Seq (pooled) | 7 |
| RMLV8 | Liver | Mary Pool | Western Australia | Australia | M | Aug-18 | Inverse rRNA RNA-Seq (pooled) | 7 |
| RMLV9 | Liver | Mary Pool | Western Australia | Australia | M | Aug-18 | Inverse rRNA RNA-Seq (pooled) | 7 |
| RMLV10 | Liver | Mary Pool | Western Australia | Australia | M | Aug-18 | Inverse rRNA RNA-Seq (pooled) | 7 |
| RMLV11 | Liver | Mary Pool | Western Australia | Australia | M | Aug-18 | Inverse rRNA RNA-Seq (pooled) | 7 |
| RMLV12 | Liver | Mary Pool | Western Australia | Australia | M | Aug-18 | Inverse rRNA RNA-Seq (pooled) | 7 |
| RMLV13 | Liver | Port Douglas | Queensland | Australia | F | Aug-18 | Inverse rRNA RNA-Seq (pooled) | 8 |
| RMLV14 | Liver | Port Douglas | Queensland | Australia | M | Aug-18 | Inverse rRNA RNA-Seq (pooled) | 8 |
| RMLV15 | Liver | Port Douglas | Queensland | Australia | M | Aug-18 | Inverse rRNA RNA-Seq (pooled) | 8 |
| RMLV16 | Liver | Port Douglas | Queensland | Australia | M | Aug-18 | Inverse rRNA RNA-Seq (pooled) | 8 |
| RMLV17 | Liver | Port Douglas | Queensland | Australia | F | Aug-18 | Inverse rRNA RNA-Seq (pooled) | 8 |
| RMLV18 | Liver | Port Douglas | Queensland | Australia | F | Aug-18 | Inverse rRNA RNA-Seq (pooled) | 8 |
| RMLV19 | Liver | Port Douglas | Queensland | Australia | F | Aug-18 | Inverse rRNA RNA-Seq (pooled) | 8 |
| RMLV20 | Liver | Port Douglas | Queensland | Australia | F | Aug-18 | Inverse rRNA RNA-Seq (pooled) | 8 |
| RMLV21 | Liver | Port Douglas | Queensland | Australia | F | Aug-18 | Inverse rRNA RNA-Seq (pooled) | 8 |
| RMLV22 | Liver | Port Douglas | Queensland | Australia | M | Aug-18 | Inverse rRNA RNA-Seq (pooled) | 8 |
| RMLV23 | Liver | Port Douglas | Queensland | Australia | M | Aug-18 | Inverse rRNA RNA-Seq (pooled) | 8 |
| RMLV24 | Liver | Port Douglas | Queensland | Australia | F | Aug-18 | Inverse rRNA RNA-Seq (pooled) | 8 |
| E1/31 | Liver | Rossville | Queensland | Australia | F | Dec-18 | PCR screening only | N/A |
| E1/32 | Liver | Rossville | Queensland | Australia | F | Dec-18 | PCR screening only | N/A |
| E1/33 | Liver | Rossville | Queensland | Australia | F | Dec-18 | PCR screening only | N/A |
| E1/34 | Liver | Rossville | Queensland | Australia | F | Dec-18 | PCR screening only | N/A |
| E1/35 | Liver | Rossville | Queensland | Australia | F | Dec-18 | PCR screening only | N/A |
| E1/36 | Liver | Rossville | Queensland | Australia | F | Dec-18 | PCR screening only | N/A |
| E1/37A | Liver | Rossville | Queensland | Australia | M | Dec-18 | PCR screening only | N/A |
| E1/37B | Liver | Rossville | Queensland | Australia | F | Dec-18 | PCR screening only | N/A |
| E1/38 | Liver | Rossville | Queensland | Australia | F | Dec-18 | PCR screening only | N/A |
| E1/39 | Liver | Rossville | Queensland | Australia | F | Dec-18 | PCR screening only | N/A |
| E1/40 | Liver | Rossville | Queensland | Australia | F | Dec-18 | PCR screening only | N/A |
| E2/41 | Liver | Croydon | Queensland | Australia | F | Dec-18 | PCR screening only | N/A |
| E2/42 | Liver | Croydon | Queensland | Australia | F | Dec-18 | PCR screening only | N/A |
| E2/43 | Liver | Croydon | Queensland | Australia | F | Dec-18 | PCR screening only | N/A |
| E2/44 | Liver | Croydon | Queensland | Australia | F | Dec-18 | PCR screening only | N/A |
| E2/45 | Liver | Croydon | Queensland | Australia | F | Dec-18 | PCR screening only | N/A |
| E2/46 | Liver | Croydon | Queensland | Australia | F | Dec-18 | PCR screening only | N/A |
| E2/47 | Liver | Croydon | Queensland | Australia | F | Dec-18 | PCR screening only | N/A |
| E2/48 | Liver | Croydon | Queensland | Australia | F | Dec-18 | PCR screening only | N/A |
| E2/49 | Liver | Croydon | Queensland | Australia | F | Dec-18 | PCR screening only | N/A |
| E2/50A | Liver | Croydon | Queensland | Australia | M | Dec-18 | PCR screening only | N/A |
| E2/50B | Liver | Croydon | Queensland | Australia | F | Dec-18 | PCR screening only | N/A |
| E3/51 | Liver | Lucinda | Queensland | Australia | F | Dec-18 | PCR screening only | N/A |
| E3/52 | Liver | Lucinda | Queensland | Australia | F | Dec-18 | PCR screening only | N/A |
| E3/53 | Liver | Lucinda | Queensland | Australia | F | Dec-18 | PCR screening only | N/A |
| E3/54 | Liver | Lucinda | Queensland | Australia | F | Dec-18 | PCR screening only | N/A |
| E3/55 | Liver | Lucinda | Queensland | Australia | F | Dec-18 | PCR screening only | N/A |
| E3/56 | Liver | Lucinda | Queensland | Australia | F | Dec-18 | PCR screening only | N/A |
| E3/57 | Liver | Lucinda | Queensland | Australia | F | Dec-18 | PCR screening only | N/A |
| E3/58 | Liver | Lucinda | Queensland | Australia | F | Dec-18 | PCR screening only | N/A |
| E3/59 | Liver | Lucinda | Queensland | Australia | F | Dec-18 | PCR screening only | N/A |
| E3/60 | Liver | Lucinda | Queensland | Australia | F | Dec-18 | PCR screening only | N/A |
| W1/1 | Liver | Kununurra | Western Australia | Australia | F | Sep-18 | PCR screening only | N/A |
| W1/2 | Liver | Kununurra | Western Australia | Australia | F | Sep-18 | PCR screening only | N/A |
| W1/3 | Liver | Kununurra | Western Australia | Australia | F | Sep-18 | PCR screening only | N/A |
| W1/4 | Liver | Kununurra | Western Australia | Australia | F | Sep-18 | PCR screening only | N/A |
| W1/5 | Liver | Kununurra | Western Australia | Australia | F | Sep-18 | PCR screening only | N/A |
| W1/6 | Liver | Kununurra | Western Australia | Australia | F | Sep-18 | PCR screening only | N/A |
| W1/7 | Liver | Kununurra | Western Australia | Australia | F | Sep-18 | PCR screening only | N/A |
| W1/8 | Liver | Kununurra | Western Australia | Australia | F | Sep-18 | PCR screening only | N/A |
| W1/9 | Liver | Kununurra | Western Australia | Australia | F | Sep-18 | PCR screening only | N/A |
| W1/10 | Liver | Kununurra | Western Australia | Australia | F | Sep-18 | PCR screening only | N/A |
| W2/11 | Liver | Old Theda | Western Australia | Australia | F | Sep-18 | PCR screening only | N/A |
| W2/12 | Liver | Old Theda | Western Australia | Australia | F | Sep-18 | PCR screening only | N/A |
| W2/13 | Liver | Old Theda | Western Australia | Australia | F | Sep-18 | PCR screening only | N/A |
| W2/14 | Liver | Old Theda | Western Australia | Australia | F | Sep-18 | PCR screening only | N/A |
| W2/15 | Liver | Old Theda | Western Australia | Australia | F | Sep-18 | PCR screening only | N/A |
| W2/16 | Liver | Old Theda | Western Australia | Australia | F | Sep-18 | PCR screening only | N/A |
| W2/17 | Liver | Old Theda | Western Australia | Australia | F | Sep-18 | PCR screening only | N/A |
| W2/18 | Liver | Old Theda | Western Australia | Australia | F | Sep-18 | PCR screening only | N/A |
| W2/19 | Liver | Old Theda | Western Australia | Australia | F | Sep-18 | PCR screening only | N/A |
| W2/20 | Liver | Old Theda | Western Australia | Australia | F | Sep-18 | PCR screening only | N/A |
| W3/21 | Liver | Mary Pool | Western Australia | Australia | F | Sep-18 | PCR screening only | N/A |
| W3/22 | Liver | Mary Pool | Western Australia | Australia | F | Sep-18 | PCR screening only | N/A |
| W3/23 | Liver | Mary Pool | Western Australia | Australia | F | Sep-18 | PCR screening only | N/A |
| W3/24 | Liver | Mary Pool | Western Australia | Australia | F | Sep-18 | PCR screening only | N/A |
| W3/25 | Liver | Mary Pool | Western Australia | Australia | F | Sep-18 | PCR screening only | N/A |
| W3/26 | Liver | Mary Pool | Western Australia | Australia | F | Sep-18 | PCR screening only | N/A |
| W3/27 | Liver | Mary Pool | Western Australia | Australia | F | Sep-18 | PCR screening only | N/A |
| W3/28 | Liver | Mary Pool | Western Australia | Australia | F | Sep-18 | PCR screening only | N/A |
| W3/29 | Liver | Mary Pool | Western Australia | Australia | F | Sep-18 | PCR screening only | N/A |
| W3/30 | Liver | Mary Pool | Western Australia | Australia | F | Sep-18 | PCR screening only | N/A |
| S11 | Spleen | Halls Creek | Western Australia | Australia | ? | May-15 | PCR screening only | N/A |
| S12 | Spleen | Halls Creek | Western Australia | Australia | ? | May-15 | PCR screening only | N/A |
| **FRENCH GUIANA** | | | | | | | | |
| **Sample ID** | **Tissue type** | **Collection location** |  | **Country of origin** | **Sex** | **Date of collection** | **Type of sequencing performed** | **RNA-Seq library number** |
| RMF017S | Spleen | Plage de Gosselin |  | French Guiana | F | Aug-17 | Inverse rRNA RNA-Seq (pooled) | 3 |
| RMF028S | Spleen | Route des Plages Drain |  | French Guiana | F | Aug-17 | Inverse rRNA RNA-Seq (pooled) | 3 |
| RMF022S | Spleen | Plage de Montjoly |  | French Guiana | F | Aug-17 | Inverse rRNA RNA-Seq (pooled) | 3 |
| RMF031L | Liver | Plage de Gosselin |  | French Guiana | F | Nov-17 | Inverse rRNA RNA-Seq (pooled) | 3 |
| RMF033L | Liver | Plage de Gosselin |  | French Guiana | M | Nov-17 | Inverse rRNA RNA-Seq (pooled) | 3 |
| RMF020L | Liver | Plage de Montjoly |  | French Guiana | F | Aug-17 | Inverse rRNA RNA-Seq (pooled) | 3 |
| RMF034L | Liver | Plage de Gosselin |  | French Guiana | M | Nov-17 | Inverse rRNA RNA-Seq (pooled) | 3 |
| RMF043L | Liver | Past Regina Wetland |  | French Guiana | M | Nov-17 | Inverse rRNA RNA-Seq (pooled) | 5 |
| RMF045L | Liver | Plage de Montjoly |  | French Guiana | M | Nov-17 | Inverse rRNA RNA-Seq (pooled) | 5 |
| RMF047L | Liver | Plage de Montjoly |  | French Guiana | F | Nov-17 | Inverse rRNA RNA-Seq (pooled) | 5 |
| RMF049L | Liver | Plage de Montjoly |  | French Guiana | F | Nov-17 | Inverse rRNA RNA-Seq (pooled) | 5 |
| RMF050L | Liver | Plage de Montjoly |  | French Guiana | F | Nov-17 | Inverse rRNA RNA-Seq (pooled) | 5 |
| RMF010L | Liver | Plage de Montjoly |  | French Guiana | F | Aug-17 | Inverse rRNA RNA-Seq (pooled) | 5 |
| RMF041L | Liver | Eastern Rainforest Pool |  | French Guiana | M | Nov-17 | Inverse rRNA RNA-Seq (pooled) | 5 |
| RMF036L | Liver | Regina Wash |  | French Guiana | M | Nov-17 | Inverse rRNA RNA-Seq (pooled) | 5 |
| RMF032L | Liver | Plage de Gosselin |  | French Guiana | M | Nov-17 | Inverse rRNA RNA-Seq (pooled) | 6 |
| RMF035L | Liver | Plage de Gosselin |  | French Guiana | M | Nov-17 | Inverse rRNA RNA-Seq (pooled) | 6 |
| RMF046L | Liver | Plage de Montjoly |  | French Guiana | F | Nov-17 | Inverse rRNA RNA-Seq (pooled) | 6 |
| RMF038L | Liver | Rainforest Puddles |  | French Guiana | M | Nov-17 | Inverse rRNA RNA-Seq (pooled) | 6 |
| RMF044L | Liver | Plage de Montjoly |  | French Guiana | F | Nov-17 | Inverse rRNA RNA-Seq (pooled) | 6 |
| RMF048L | Liver | Plage de Montjoly |  | French Guiana | F | Nov-17 | Inverse rRNA RNA-Seq (pooled) | 6 |
| RMF040L | Liver | Eastern Rainforest Pool |  | French Guiana | M | Nov-17 | Inverse rRNA RNA-Seq (pooled) | 6 |
| RMF042L | Liver | Past Regina Wetland |  | French Guiana | M | Nov-17 | Inverse rRNA RNA-Seq (pooled) | 6 |
| RMF037L | Liver | Regina Wash |  | French Guiana | M | Nov-17 | Inverse rRNA RNA-Seq (pooled) | 6 |
| RMF039L | Liver | Eastern Rainforest Pool |  | French Guiana | M | Nov-17 | Inverse rRNA RNA-Seq (pooled) | 6 |
| **HAWAI’I** | | | | | | | | |
| **Sample ID** | **Tissue type** | **Collection location** |  | **Country of origin** | **Sex** | **Date of collection** | **Type of sequencing performed** | **RNA-Seq library number** |
| RMH006S | Spleen | Haiku Gardens |  | USA (Hawaii) | F | Jun-15 | Inverse rRNA RNA-Seq (pooled) | 2 |
| RMH025S | Spleen | Kapolei Regional Park |  | USA (Hawaii) | F | Jun-15 | Inverse rRNA RNA-Seq (pooled) | 2 |
| RMH024S | Spleen | Kapolei Regional Park |  | USA (Hawaii) | F | Jun-15 | Inverse rRNA RNA-Seq (pooled) | 2 |
| RMH049S | Spleen | Haiku Gardens |  | USA (Hawaii) | F | Jun-15 | Inverse rRNA RNA-Seq (pooled) | 2 |
| RMH050S | Spleen | Haiku Gardens |  | USA (Hawaii) | F | Jun-15 | Inverse rRNA RNA-Seq (pooled) | 2 |
| RMH018S | Spleen | Kapolei Regional Park |  | USA (Hawaii) | F | Jun-15 | Inverse rRNA RNA-Seq (pooled) | 2 |
| RMH008S | Spleen | Haiku Gardens |  | USA (Hawaii) | F | Jun-15 | Inverse rRNA RNA-Seq (pooled) | 2 |
| RMH043S | Spleen | Haiku Gardens |  | USA (Hawaii) | F | Jun-15 | Inverse rRNA RNA-Seq (pooled) | 2 |
| RMH020S | Spleen | Kapolei Regional Park |  | USA (Hawaii) | F | Jun-15 | Inverse rRNA RNA-Seq (pooled) | 2 |
| RMH021S | Spleen | Kapolei Regional Park |  | USA (Hawaii) | F | Jun-15 | Inverse rRNA RNA-Seq (pooled) | 2 |
|  |  |  |  |  |  |  |  |  |

| **Sample ID** | **Tissue type** | **Collection location** |  | **Country of origin** | **Sex** | **Date of collection** | **Type of sequencing** |
| --- | --- | --- | --- | --- | --- | --- | --- |
| RMF031L | Liver | Plage de Gosselin |  | French Guiana | F | Nov-17 | Genomic DNA-Seq |
| RMF042L | Liver | Past Regina Wetland |  | French Guiana | M | Nov-17 | Genomic DNA-Seq |
| RMF044L | Liver | Plage de Montjoly |  | French Guiana | F | Nov-17 | Genomic DNA-Seq |
| RMF048L | Liver | Plage de Montjoly |  | French Guiana | F | Nov-17 | Genomic DNA-Seq |

Table S1b: Samples used for DNA-sequencing only

Table S1c: Previously generated RNA-Seq datasets used in this study

| **Sample ID** | **Tissue type** | **Collection location** | **Country of origin** | **Sex** | **Date of collection** | **Type of sequencing performed** | **SRA ID** |
| --- | --- | --- | --- | --- | --- | --- | --- |
| RMF010S | Spleen | Plage de Montjoly | French Guiana | F | Aug-17 | poly(A) RNA-Seq | SRR8334545 |
| RMF047S | Spleen | Plage de Montjoly | French Guiana | F | Nov-17 | poly(A) RNA-Seq | SRR8334537 |
| RMF048S | Spleen | Plage de Montjoly | French Guiana | F | Nov-17 | poly(A) RNA-Seq | SRR8334540 |
| RMF049S | Spleen | Plage de Montjoly | French Guiana | F | Nov-17 | poly(A) RNA-Seq | SRR8334542 |
| RMF022S | Spleen | Plage de Montjoly | French Guiana | F | Aug-17 | poly(A) RNA-Seq | SRR8334546 |
| RMF028S | Spleen | Route des Plages Drain | French Guiana | F | Aug-17 | poly(A) RNA-Seq | SRR8334538 |
| RMF017S | Spleen | Plage de Gosselin | French Guiana | F | Aug-17 | poly(A) RNA-Seq | SRR8334541 |
| RMF046S | Spleen | Plage de Montjoly | French Guiana | F | Nov-17 | poly(A) RNA-Seq | SRR8334539 |
| RMH049S | Spleen | Haiku Gardens | USA (Hawaii) | F | Jun-15 | poly(A) RNA-Seq | SRR8334535 |
| RMH025S | Spleen | Kapolei Regional Park | USA (Hawaii) | F | Jun-15 | poly(A) RNA-Seq | SRR8334551 |
| RMH043S | Spleen | Haiku Gardens | USA (Hawaii) | F | Jun-15 | poly(A) RNA-Seq | SRR8334547 |
| RMH050S | Spleen | Haiku Gardens | USA (Hawaii) | F | Jun-15 | poly(A) RNA-Seq | SRR8334548 |
| RMH021S | Spleen | Kapolei Regional Park | USA (Hawaii) | F | Jun-15 | poly(A) RNA-Seq | SRR8334549 |
| RMH024S | Spleen | Kapolei Regional Park | USA (Hawaii) | F | Jun-15 | poly(A) RNA-Seq | SRR8334550 |
| RMH006S | Spleen | Haiku Gardens | USA (Hawaii) | F | Jun-15 | poly(A) RNA-Seq | SRR8334552 |
| RMH008S | Spleen | Haiku Gardens | USA (Hawaii) | F | Jun-15 | poly(A) RNA-Seq | SRR8334544 |
| RMH020S | Spleen | Kapolei Regional Park | USA (Hawaii) | F | Jun-15 | poly(A) RNA-Seq | SRR8334543 |
| RMH018S | Spleen | Kapolei Regional Park | USA (Hawaii) | F | Jun-15 | poly(A) RNA-Seq | SRR8334536 |
| RHIMB_LIVER | Liver | Commercial supplier | Denmark | Unknown | Unknown | Inverse rRNA RNA-Seq | ERR2198610 |

**Table S2: Primers used to amplify virus-like transcripts from cane toad tissues with PCR/RT-PCR.**

| **Virus** | **Library/sample in which sequence is present** | **PCR target region** | **Primer pair used for screening** | **Amplicon length (bp)** |
| --- | --- | --- | --- | --- |
| Cayenne cane toad picornavirus | RM_3, RM_6 | VP2 | MML198F | 153 |
|  |  |  | 5’-CGACACTACCTTTCTAATCC-3’ |  |
|  |  |  | MML199R |  |
|  |  |  | 5’-AAGAGTGAGTTGCCAAGG-3’ |  |
| R. marina erythrocytic-like iridovirus | RM_6 | Major capsid | MCPF | 530 |
|  |  |  | 5’-CGAAGCGGCTATGAGACCAT-3’ |  |
|  |  |  | MCPR |  |
|  |  |  | 5’-GCCGTCGTCCAATTAGACCA-3’ |  |
| Cane toad hepe-like virus* | RM_8 | Capsid | MML192F | 162 |
|  |  |  | 5’-GTACTCCACGCTTATTCC-3’ |  |
|  |  |  | MML193R |  |
|  |  |  | 5’- CTTGTAATTCAGATCTCTGC-3’ |  |
| Cane toad nackedna-like virus* | RMF048L | P/polymerase | MML230F | 161 |
|  |  |  | 5’-CCTGGGAAGGGCTTTCTCAA-3’ |  |
|  |  |  | MML231R |  |
|  |  |  | 5’-TGATTGCATGTCCCATGGGT-3’ |  |
| Rhimavirus-A | RM_1, RM_7 | 5’-UTR | MML157 | 304 |
|  |  |  | 5’-GGATCTTTCCTCTTTATGAGC-3’ |  |
|  |  |  | MML158 |  |
|  |  |  | 5’-GGCATTCCTCATATTTGACTCC-3‘ |  |
| *target sequences were unable to be amplified with Standard *Taq* Polymerase (NEB) and were instead amplified with Platinum™ SuperFi™ DNA Polymerase (Thermo Fisher) | | | | |

**Table S3a: Primers used for PCR amplification and Sanger sequencing of unknown sequence from novel cane toad viruses – Cayenne cane toad picornavirus.**

| **Primer ID** | **Primer sequence** | **Primer orientation** | **Target genome region** |
| --- | --- | --- | --- |
| MML198 | CGACACTACCTTTCTAATCC | F | VP0 |
| MML199 | AAGAGTGAGTTGCCAAGG | R | VP0 |
| MML200 | ACAGTCGTTTTGTTGAAGG | F | 2C |
| MML201 | CAAGTGTGAAACGTAACG | R | 2C |
| MML202 | GAGAAGCGAAGTGAATACC | R | 3C |
| MML203 | GGATCTGATACGTTATCTCC | F | 3C |
| MML204 | GTACGCTTCACAAGGTAGG | F | 3D |
| MML205 | CCTCCACAACTCTAGTCG | R | 3D |
| MML206 | TCTGGAACTTGAATCACG | F | 3D |
| MML207 | GTTTGTAGACGTGGATGG | R | 3D |
| MML208 | GCTCAAGGTCCGAATCCACA | R | 3D |
| MML209 | CAGCCTCCAAAACTGGGGAT | F | 3D |
| MML210 | CAGGCTTGTGCAGAGCAAAG | F | 2C |
| MML211 | CAAAACGACTGTCCGCTTCG | R | 2C |
| MML212 | CACAGTCGACAGGTCAGACC | F | VP2 |
| MML213 | GGGTGGGAAAAGCAGTACCA | R | VP2 |
| MML214 | GTATCTACCCACGCCTGAGC | F | 3D |
| MML215 | CTTCTCGACAGCACGGAGTT | R | 3D |
| MML216 | GAGAAAGCTTTGAAAGAGC | F | 2C |
| MML217 | ACTATGACCTTGGGTTAGG | R | 3B/3C |
| MML218 | ACCGGCAACCTCAACATCATTG | R | VP1 |
| MML219 | TATCTCTGATGCTGGCGTCACG | F | VP1 |
| MML220 | ATCATGCCACACCGGAGATG | F | 2B |
| MML221 | CCATCTTGTTTCCGCCATGC | F | VP1 |
| MML232 | GGTATGGCGTCACAGGTTGA | F | VP3 |
| MML233 | GCTTCATTTGCGCAGATGGT | F | 2C |
| MML234 | TCTGATGTGTGGGCTGGTTC | F | VP1 |
| MML235 | CTTTTGGCGCTACACGTGTC | F | 2C |
| MML236 | GACGTTGGTGTAAGAGCCGA | F | VP1 |
| MML237 | ACGGCCAGGTTTTCTGTAGC | R | 2C |
| MML240 | TAGCCAGCTGCAGTTAACGT | F | 5'-UTR |
| MML241 | AAACCAACCACGCTTGATGC | R | VP0 |
| MML242 | TGGACGGATCATCCAGAGGT | R | VP0 |
| MML253 | ATAGTGTGACCACCGCCTTG | F | 2B |
| MML254 | AAGCTTCACGATCAGCTGCT | F | 2B |
| MML267 | GTTCTTTTCCTGGCTGCTGCT | F | VP0 |
| MML268 | GCACATCAACTTGCTGCACA | R | VP3 |
| MML281 | ACCCAAAGCGGTAGAAAGGG | R | VP0 |
| MML282 | GGTCTGACCTGTCGACTGTG | R | VP0 |
| MML315 | TCAAGATGAGCTGGGCCTTG | F | VP0 |
| MML316 | TGTGCAGCAAGTTGATGTGC | F | VP3 |
| MML317 | ACGCCAGCATCAGAGATAGC | R | VP1 |
| MML318 | ATTTTTCATCCCCCGCCCAT | F | VP1 |
| MML319 | GCGAACCAGCACCACAATTT | R | 2B |

Table S3b: Primers used for PCR amplification and Sanger sequencing of unknown sequence from novel cane toad viruses: Rhinella marina papillomavirus 1.

| **Primer ID** | **Primer sequence** | **Primer orientation** | **Target genome region** |
| --- | --- | --- | --- |
| MML273 | GGCTTCCTCCTGGAACCTTC | F | L1 |
| MML274 | CATCGGAACCCTCAGCAGTT | F | L1 |
| MML275 | GGCTTGCTTCGACTTCACAAG | R | L1 |
| MML276 | GCTTTGGCTTGCTTCGACTT | R | L1 |
| MML277 | AATCTAGGCGCTCCAGAAGG | F | L1 |
| MML278 | GGCCCAAGTTCAACCAATGG | F | L1 |
| MML279 | TGTGCCTGGACAATTCCTTCA | R | L1 |
| MML280 | GCCTGGACAATTCCTTCAGGA | R | L1 |
| MML259 | ATAGGGATGGGCTCTGGGTT | F | L1 |
| MML260 | TTTCAACAAGAGCTGGGCCTA | R | L1 |
| MML261 | CCACTAGATCCCCGTCCTCA | F | L1 |
| MML262 | AACCCAGAGCCCATCCCTAT | R | L1 |
| MML263 | CTCATGGCCCACTGAACCAT | F | L1 |
| MML264 | ATAAGGTCCAGGGGCCTGAT | R | L1 |
| MML265 | ATCAGGCCCCTGGACCTTAT | F | L1 |
| MML266 | CCAAATGTTGTGCCGATCCC | R | L1 |

**Table S4: Annotation of iridovirus genes from the RM_6 dataset encoded by the genome of Rhinella marina erythrocytic-like virus.**

| **ORF ID** | **Transcript ID (Rhinella marina erythrocytic-like iridovirus)** | **Protein length (aa)** | **Predicted gene encoded by this ORF** | **ORF status** | **closest viral match [virus of origin]** | **E-value to closest match** | **% aa identity to closest match** |
| --- | --- | --- | --- | --- | --- | --- | --- |
| RMELV_32 | DN37299_c0_g2_i1 | 466 | Major capsid protein |  | major capsid protein, partial [Erythrocytic necrosis virus] | 0.00E+00 | 75.30% |
| RMELV_33 | DN37390_c0_g2_i1 | 993* | Hypothetical protein RMELV033 |  | hypothetical protein, partial [Erythrocytic necrosis virus] | 0.00E+00 | 48.10% |
| RMELV_42 | DN51760_c0_g1_i1 | 860 | D5 Family NTPase involved in DNA replication |  | D5 family NTPase ATPase [Shrimp hemocyte iridescent virus] | 0.00E+00 | 46.50% |
| RMELV_64 | DN66899_c0_g1_i1 | 1027 | DNA dependent RNA polymerase II second largest subunit |  | RNA polymerase beta subunit [Lymphocystis disease virus Sa] | 0.00E+00 | 39.20% |
| RMELV_66 | DN90760_c0_g1_i1 | 1202 | DNA dependent RNA polymerase II largest subunit |  | 097L [Cherax quadricarinatus iridovirus] | 0.00E+00 | 41.70% |
| RMELV_67 | DN9175_c0_g1_i1 | 939 | DNA polymerase family B exonuclease |  | putative DNA polymerase, partial [Erythrocytic necrosis virus] | 0.00E+00 | 62.00% |
| RMELV_4 | DN13902_c0_g1_i1 | 898 | NTPase I |  | 004L [Cherax quadricarinatus iridovirus] | 4.48E-159 | 33.50% |
| RMELV_24 | DN28191_c0_g1_i1 | 299 | Putative replication factor and/or DNA binding/packaging |  | putative DNA binding/packing protein, partial [Erythrocytic necrosis virus] | 2.40E-147 | 68.80% |
| RMELV_6 | DN140020_c0_g1_i1 | 516 | Papain-like proteinase |  | 103L [Cherax quadricarinatus iridovirus] | 6.13E-135 | 46.50% |
| RMELV_14 | DN25218_c0_g1_i2 | 398 | Ribonucleotide reductase small subunit |  | Ribonucleotide reductase [Lymphocystis disease virus - isolate China] | 3.31E-125 | 52.90% |
| RMELV_55 | DN53483_c0_g1_i1 | 967* | Putative tyrosine kinase/LPS-modifying enzyme |  | 061L [Cherax quadricarinatus iridovirus] | 4.21E-125 | 32.70% |
| RMELV_13 | DN20703_c0_g1_i1 | 336 | DNA repair protein/FLAP endonuclease |  | putative DNA repair protein, partial [Erythrocytic necrosis virus] | 4.98E-118 | 53.40% |
| RMELV_41 | DN50679_c0_g1_i1 | 613 | Putative DNA double-strand break repair rad50 ATPase-like protein |  | putative DNA double-strand break repair rad50 ATpase-like protein [Erythrocytic necrosis virus] | 5.52E-118 | 36.90% |
| RMELV_37 | DN44463_c0_g1_i2 | 392 | Immediate early protein ICP-46/RNA ligase |  | putative immediate early ICP-46 [Erythrocytic necrosis virus] | 8.66E-115 | 57.50% |
| RMELV_65 | DN89206_c0_g1_i1 | 398 | Hypothetical protein RMELV065 |  | hypothetical protein, partial [Erythrocytic necrosis virus] | 1.59E-102 | 41.70% |
| RMELV_27 | DN29642_c0_g1_i1 | 321 | Myristylated membrane protein |  | putative myristylated membrane protein, partial [Erythrocytic necrosis virus] | 5.79E-91 | 46.70% |
| RMELV_16 | DN26284_c0_g1_i1 | 315 | DNA exonuclease repair subunit |  | hypothetical protein [Erythrocytic necrosis virus] | 1.21E-86 | 47.90% |
| RMELV_63 | DN64728_c0_g1_i1 | 345 | Helicase-like protein |  | putative helicase-like protein [Erythrocytic necrosis virus] | 1.95E-86 | 47.50% |
| RMELV_69 | DN139502_c0_g1_i1 | 223 | Cytosine DNA methyltransferase |  | cytosine DNA methyltransferase [Turbot reddish body iridovirus] | 9.61E-86 | 59.50% |
| RMELV_20 | DN28023_c0_g1_i1 | 245 | ATPase |  | 032R [Cherax quadricarinatus iridovirus] | 2.80E-80 | 48.80% |
| RMELV_71 | DN140734_c0_g1_i1 | 375 | DNA repair exonuclease SbcCD D subunit |  | putative DNA repair exonuclease SbcCD D subunit [Erythrocytic necrosis virus] | 4.99E-73 | 37.50% |
| RMELV_19 | DN28023_c0_g1_i1 | 267 | Hypothetical protein RMELV019 |  | hypothetical protein, partial [Erythrocytic necrosis virus] | 7.79E-67 | 47.00% |
| RMELV_22 | DN28075_c0_g1_i1 | 277* | Serine/threonine protein kinase |  | putative serine/threonine protein kinase, partial [Erythrocytic necrosis virus] | 5.01E-64 | 43.90% |
| RMELV_34 | DN41703_c0_g1_i1 | 325* | Helicase family |  | hypothetical protein, partial [Erythrocytic necrosis virus] | 2.06E-63 | 72.30% |
| RMELV_46 | DN52778_c0_g1_i3 | 438 | Putative phosphotransferase |  | putative phosphotransferase, partial [Erythrocytic necrosis virus] | 5.37E-61 | 37.30% |
| RMELV_8 | DN17870_c0_g1_i1 | 267 | Proliferating cell nuclear antigen |  | putative proliferating cell nuclear antigen, partial [Erythrocytic necrosis virus] | 1.72E-58 | 50.00% |
| RMELV_50 | DN53483_c0_g4_i1 | 325 | Hypothetical protein RMELV050 |  | hypothetical protein [Erythrocytic necrosis virus] | 1.19E-57 | 42.90% |
| RMELV_30 | DN37299_c0_g1_i1 | 173 | Hypothetical protein RMELV030 |  | hypothetical protein [Erythrocytic necrosis virus] | 5.25E-54 | 53.40% |
| RMELV_10 | DN20563_c0_g1_i1 | 379 | Putative phosphotransferase |  | putative phosphotransferase [Erythrocytic necrosis virus] | 4.98E-41 | 41.00% |
| RMELV_49 | DN53392_c0_g1_i3 | 275 | Patatin-like phospholipase |  | 047L [Cherax quadricarinatus iridovirus] | 1.67E-39 | 32.40% |
| RMELV_57 | DN53483_c0_g3_i1 | 188 | Putative deoxynucleoside kinase |  | putative deoxynucleoside kinase, partial [Erythrocytic necrosis virus] | 2.35E-39 | 36.60% |
| RMELV_56 | DN53483_c0_g3_i1 | 180 | Putative deoxynucleoside kinase |  | putative deoxynucleoside kinase, partial [Erythrocytic necrosis virus] | 4.18E-38 | 37.30% |
| RMELV_29 | DN37299_c0_g1_i1 | 192 | Haloacid dehalogenase-like hydrolase |  | hypothetical protein, partial [Erythrocytic necrosis virus] | 1.01E-37 | 65.90% |
| RMELV_5 | DN140020_c0_g1_i1 | 257 | Lysozyme g-like homolog |  | lysozyme G-like 2 S homeolog precursor [Xenopus laevis] | 1.22E-37 | 39.40% |
| RMELV_70 | DN63151_c2_g3_i1 | 266 | p31k protein |  | hypothetical protein, partial [Erythrocytic necrosis virus] | 1.12E-36 | 49.10% |
| RMELV_61 | DN56615_c9_g2_i4 | 78 | Transcription elongation factor TFIIS |  | putative transcription elongation factor SII [Erythrocytic necrosis virus] | 2.32E-29 | 65.80% |
| RMELV_1 | DN116043_c0_g1_i1 | 260 | Ribonuclease III |  | RNase III [Singapore grouper iridovirus] | 7.60E-29 | 56.90% |
| RMELV_7 | DN141144_c0_g1_i1 | 139 | ERV1/Air family |  | 160L [Cherax quadricarinatus iridovirus] | 1.93E-27 | 40.30% |
| RMELV_39 | DN50679_c0_g0_i1 | 340 | Eukaryotic-like serine-threonine protein kinase |  | PREDICTED: serine/threonine-protein kinase VRK2-like [Elephantulus edwardii] | 2.54E-25 | 30.80% |
| RMELV_15 | DN25218_c0_g1_i2 | 145 | dUTPase |  | dUTP pyrophosphatase [Pigeonpox virus] | 1.00E-24 | 38.40% |
| RMELV_44 | DN52486_c0_g4_i1 | 425 | Serine/threonine protein kinase |  | serine/threonine protein kinase [Flamingopox virus FGPVKD09] | 1.36E-24 | 31.90% |
| RMELV_11 | DN20703_c0_g1_i1 | 429 | Putative XPPG-RAD2 type nuclease |  | 056R [Cherax quadricarinatus iridovirus] | 8.55E-24 | 21.80% |
| RMELV_72 | DN101420_c0_g1_i1 | 164* | US22 family protein |  | US22 family protein [Frog virus 3] | 1.01E-21 | 37.40% |
| RMELV_68 | DN69916_c0_g1_i1 | 433 | Myristylated membrane protein |  | Hypothetical protein ORF088L (Grouper iridovirus) | 1.09E-17 | 24.50% |
| RMELV_59 | DN53483_c0_g3_i1 | 153 | RuVC-like Holliday junction resolvase |  | 152R [Cherax quadricarinatus iridovirus] | 1.36E-13 | 37.40% |
| RMELV_12 | DN20703_c0_g1_i1 | 247 | High mobility group protein homolog |  | 176L [Cherax quadricarinatus iridovirus] | 1.30E-11 | 31.80% |
| RMELV_3 | DN118158_c0_g1_i1 | 259 | Uvr/REP helicase |  | putative Uvr/REP helicase, partial [Erythrocytic necrosis virus] | 6.68E-10 | 59.60% |
| RMELV_62 | DN64728_c0_g1_i1 | 159 | Hypothetical protein RMELV062 |  | hypothetical protein BOX15_Mlig020667g1, partial [Macrostomum lignano] | 1.60E-09 | 34.40% |
| RMELV_25 | DN29642_c0_g1_i1 | 321 | E3 ubiquitin-protein ligase-like protein |  | E3 ubiquitin-protein ligase CIP8-like [Carica papaya] | 5.32E-09 | 44.80% |
| RMELV_35 | DN44463_c0_g1_i2 | 133* | Hypothetical protein RMELV035 |  | WD repeat-containing protein, putative [Hepatocystis sp. ex Piliocolobus tephrosceles] | 1.94E-08 | 53.50% |
| RMELV_38 | DN50679_c0_g0_i1 | 181 | Hypothetical protein RMELV038 |  | hypothetical protein Hyperionvirus1_11 [Hyperionvirus sp.] | 1.04E-07 | 28.60% |
| RMELV_60 | DN56615_c9_g2_i4 | 97 | Hypothetical membrane-like protein RMELV060 |  | putative membrane protein [Emiliania huxleyi virus 99B1] | 1.57E-02 | 54.30% |
| RMELV_21 | DN28023_c0_g1_i1 | 169 | Hypothetical protein RMELV021 |  | hypothetical protein ECIV_ORF42 [European chub iridovirus] | 9.70E-02 | 33.70% |
| RMELV_36 | DN44463_c0_g1_i2 | 126* | Hypothetical protein RMELV036 |  | hypothetical protein [Rickettsiales bacterium] | 1.04E-01 | 54.80% |
| RMELV_54 | DN53483_c0_g4_i1 | 294 | Hypothetical protein RMELV054 |  | hypothetical protein [Methanosarcina barkeri] | 2.10E-01 | 59.00% |
| RMELV_26 | DN29642_c0_g1_i1 | 151* | Hypothetical protein with RING finger domain RMELV026 |  | hypothetical protein jhhlp_001452 [Lomentospora prolificans] | 8.26E-01 | 41.10% |
| RMELV_47 | DN53392_c0_g1_i3 | 91 | Hypothetical protein RMELV047 |  | hypothetical protein [Gammaproteobacteria bacterium] | 9.47E-01 | 25.60% |
| RMELV_28 | DN37299_c0_g1_i1 | 59 | Hypothetical protein RMELV028 |  | DUF726 domain-containing protein [Arcobacter cryaerophilus] | 3.98E+00 | 39.00% |
| RMELV_53 | DN53483_c0_g4_i1 | 67 | Hypothetical protein RMELV053 |  | hypothetical protein OCBIM_22029485mg [Octopus bimaculoides] | 5.83E+00 | 31.10% |
| RMELV_9 | DN20563_c0_g1_i1 | 594 | Hypothetical protein RMELV009 |  | [hypothetical protein [Firmicutes bacterium]](https://blast.ncbi.nlm.nih.gov/Blast.cgi#alnHdr_NLN84099) | 7.85E+00 | 39.50% |
| RMELV_2 | DN118158_c0_g1_i1 | 17* | Hypothetical protein RMELV002 |  | No match in nr database |  |  |
| RMELV_17 | DN28023_c0_g1_i1 | 154 | Hypothetical protein RMELV017 |  | No match in nr database |  |  |
| RMELV_18 | DN28023_c0_g1_i1 | 143 | Hypothetical protein RMELV018 |  | No match in nr database |  |  |
| RMELV_23 | DN28191_c0_g1_i1 | 183 | Hypothetical protein RMELV023 |  | No match in nr database |  |  |
| RMELV_31 | DN37299_c0_g2_i1 | 132 | Hypothetical protein RMELV031 |  | No match in nr database |  |  |
| RMELV_40 | DN50679_c0_g0_i1 | 170 | Hypothetical protein RMELV040 |  | No match in nr database |  |  |
| RMELV_43 | DN52486_c0_g4_i1 | 238 | Hypothetical protein RMELV043 |  | No match in nr database |  |  |
| RMELV_45 | DN52778_c0_g1_i3 | 305 | Hypothetical protein RMELV045 |  | No match in nr database |  |  |
| RMELV_48 | DN53392_c0_g1_i3 | 591 | Hypothetical protein RMELV048 |  | No match in nr database |  |  |
| RMELV_51 | DN53483_c0_g4_i1 | 159 | Hypothetical protein RMELV051 |  | No match in nr database |  |  |
| RMELV_52 | DN53483_c0_g4_i1 | 213 | Hypothetical protein RMELV052 |  | No match in nr database |  |  |
| RMELV_58 | DN53483_c0_g3_i1 | 328 | Hypothetical protein RMELV058 |  | No match in nr database |  |  |
|  | 39 |  |  |  |  |  |  |
|  | Core Iridoviridae conserved gene |  |  |  |  |  |  |
|  | Match to another iridovirus protein | |  |  |  |  |  |
|  | Match to another large nucleocytoplasmic DNA virus protein | | |  |  |  |  |
|  | Match to eukaryotic protein |  |  |  |  |  |  |
|  | No match in nr database |  |  |  |  |  |  |
| *full protein coding ORF is not represented | |  |  |  |  |  |  |
